# Supplementary figures and images for: Feasibility of working with a wholesale supplier to co-design and test acceptability of an intervention to promote smaller portions: an uncontrolled before-and-after study in British Fish & Chip shops
Source: BMJ Open. 2019 Feb 6;9(2):e023441. doi: 10.1136/bmjopen-2018-023441 (PMC6377521; doi:10.1136/bmjopen-2018-023441)

## Supplementary File A: Logic model

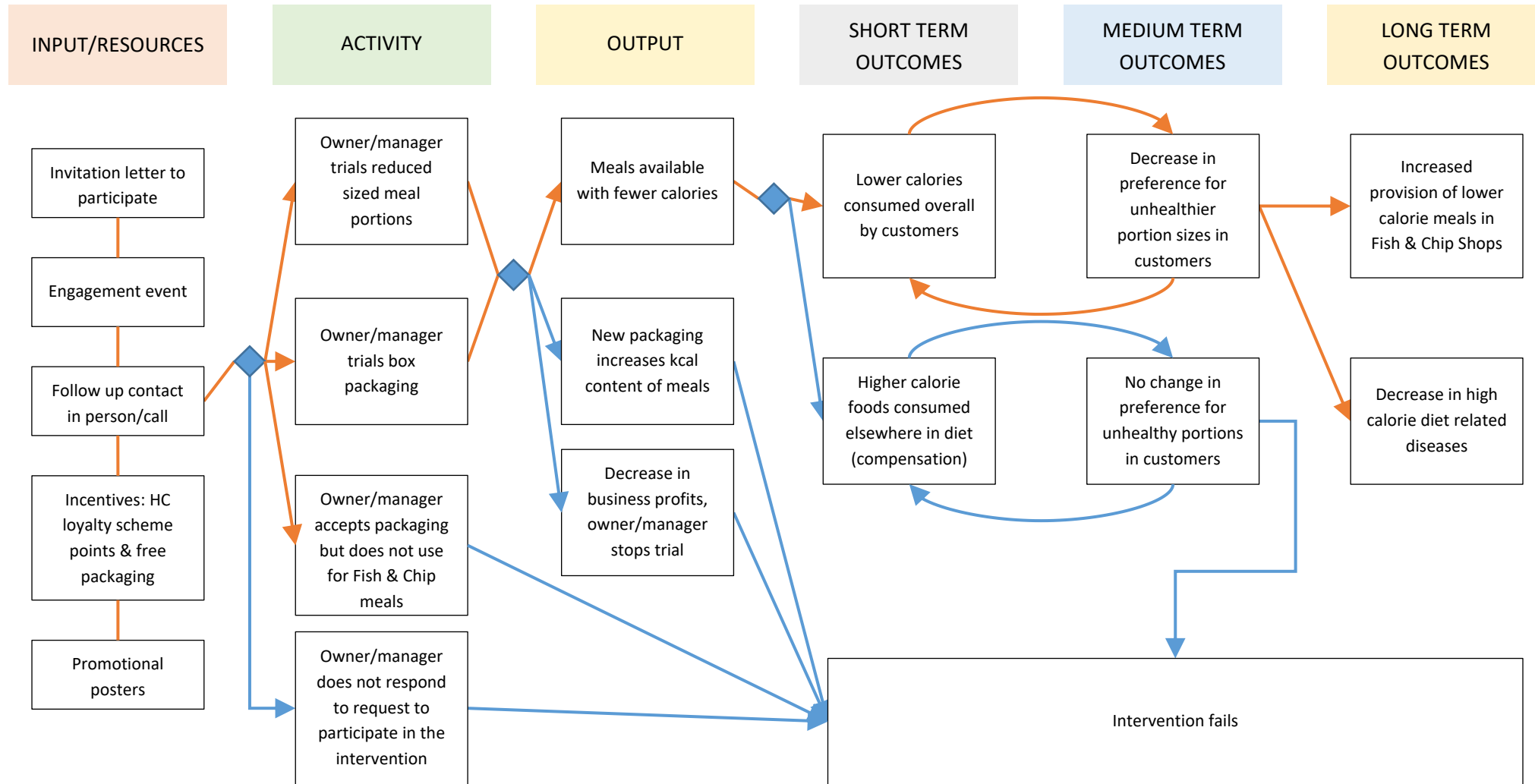

Supplement: Supplementary data [file bmjopen-2018-023441supp001.pdf]
