# Supplementary material for: Feasibility of working with a wholesale supplier to co-design and test acceptability of an intervention to promote smaller portions: an uncontrolled before-and-after study in British Fish & Chip shops
Source: BMJ Open. 2019 Feb 6;9(2):e023441. doi: 10.1136/bmjopen-2018-023441 (PMC6377521; doi:10.1136/bmjopen-2018-023441)
Supplement: Supplementary data [file bmjopen-2018-023441supp002.pdf]

Supplementary File B: Goal-setting form

Team Spirit Event, Henry Colbeck Limited,

Wednesday 6th April 2016

Business name: ____

Business representatives:

Name: ____ Role: owner/ manager/ staff (circle answer)

Name: ____ Role: owner/ manager/ staff (circle answer)

From the topics covered at this Team Spirit event; what are the things you could change in your business?

	1. Could your business make this change? (Y/N)	2. Are you willing to try this change? (Y/N)
**Change to structured packaging:** *e.g. introduce bio boxes*		
**Make smaller portions available to your *customers* at *all times***		

Public Pledge: *Detail what, which and when you will change*

**What to change?** *Structured packaging /Smaller portions*	**Which packaging will you use for which change?** *Single compartment corrugated cardboard box/Double compartment corrugated cardboard box/Bio box/MK*	**When will you do it?** *Date*	**How sure (confident) are you that your business can achieve this?** *From 1-5 (1=not at all sure; 5= very sure)**
1.			
2.			

*1= not at all sure; 2= not very sure; 3= neither; 4= somewhat sure; 5= very sure

I give consent to be contacted both, by phone, or in person by Newcastle University to provide them with details of the:

- Packaging changes I have made (if any)
- The number of Fish & Chips portions (all sizes) sold before and after the project
- My comments and opinions of the Team Spirit event
- My comment and opinions of any changes I have made following the event

Additionally, I will allow Newcastle University to speak with a few of my customers to hear their thoughts.

Signed_________________________________ Print Name_____________________________________

Phone Number (and best time to call) ____

Packaging key:

- Corrugated cardboard boxes are available in either single or double compartment versions. The double compartment boxes provides separate compartments for each of the Fish & Chip meal components.
- The Bio box packaging are a single compartment box constructed from biodegradable material extracted from sugar cane.
- MK packaging are a single compartment box constructed of non-corrugated food grade cardboard.
