# Supplementary material for: Feasibility of working with a wholesale supplier to co-design and test acceptability of an intervention to promote smaller portions: an uncontrolled before-and-after study in British Fish & Chip shops
Source: BMJ Open. 2019 Feb 6;9(2):e023441. doi: 10.1136/bmjopen-2018-023441 (PMC6377521; doi:10.1136/bmjopen-2018-023441)
Supplement: Supplementary data [file bmjopen-2018-023441supp003.pdf]

**Supplementary File C: TIDieR checklist**

Description of the portion control engagement event using the template for intervention description and replication (TIDieR) checklist.[1]

| | |
|---|---|
| 1. Name [page 8] | Engagement event – Henry Colbeck Ltd |
| 2. Rationale, theory, goal [page 8] | Fish & Chip meals from takeaways are generally characterised by large portion sizes.[2] People who eat more meals away from home tend to eat more calories per day [3] and consumption of takeaway meals has been linked to weight gain [4] and diet-related diseases.[5] A systematic review revealed that a person consumes more when presented with a larger-sized portion.[6]<br><br>The behaviour change techniques [7 8] used during the training were:<br>• Providing information on behavioural outcomes<br>• Instruction on how to perform the behaviour<br>• Demonstration of behaviour/credible source/vicarious consequences<br>• Information about others' approval<br>• Pros and cons<br>• Behavioural practice/rehearsal<br>• SMART goal setting (behaviour) and action planning<br>• Behavioural contract<br>• Adding objects to the environment<br>• Self-monitoring of behaviour (sales)<br>• Monitoring of behaviour by others without feedback<br>• Reward (outcome)/Material Incentive<br>• Financial incentive/form for specific goods [8]<br><br>Aim of the intervention: To increase the provision and promotion of smaller portion meals available to all customers throughout all opening hours and/or increased/renewed focus on portion control across all meals. |
| 3. Materials [page 8] | Information provision on the consequences of behaviour and behaviour change opportunities, presented by owners of established Fish & Chip Shops and the supplier.<br><br>Owners, managers and their staff of Fish & Chip Shops that attended were presented with details, primarily financial, of the benefits of effective portion control and the promotion and provision of smaller portion meals. These were predominantly in the form of PowerPoint presentations, but also included further visual aids such as the displaying of a locally purchased Fish & Chips meal on an 11 inch plate to illustrate the volume of food delivered. |

| | |
|---|---|
| | Participants were asked to read and sign a 'public pledge' as part of the goal setting form that stipulated which proposed activities they could and would be willing to make in their business.<br><br>Owners/managers were offered, by the supplier, two incentives to support fulfilment of the pledges. These were loyalty scheme points (financial) and a free case (100 units) of box packaging of their choosing (specific goods). Additionally, they were offered free posters to promote the availability of the smaller portion meals to the shop's customers.<br><br>The research team also provide a booklet to record Fish & Chip meal sales during the evaluation period to monitor sales behaviour.<br><br>A free lunch was included as part of the engagement event for all those that attended. |
| 4. Procedures [page 8] | Fish & Chip Shop owners/managers were invited via a postal communication from the supplier one month prior to the engagement event.<br><br>On the day of the event, participants were invited to a hotel in close proximity (under 3km) to Henry Colbeck Ltd's office and warehouse facility.<br><br>There was a range of presentations delivered from both the perspective of the supplier, Henry Colbeck Ltd and Fish & Chip business owners, as well as study data collection requirements from the research team (LG).<br><br>Henry Colbeck Ltd's presentations set out the reasons for initiating the event, and contextualised existing portion sizes in the Fish & Chip industry in relation to both an adult's diet as well as other sectors of the fast-food industry. They detailed the packaging available to support improved portion control and smaller meal portion delivery. They also stated the items that they would provide to incentivise shop participation.<br><br>One business owner (Owner A) detailed his experience of delivering highly regulated portion sizes, as well as the benefits to his business of providing a range of smaller portion meals. This included knowing one's target portion size, selecting the appropriate packaging, staff training, sales and profit margins of smaller meals. A second business owner (Owner B) detailed the experience of the implementation and financial success within his shop of smaller portion meals.<br><br>LG detailed what data the research team intended to collect as part of the evaluation.<br><br>Following the presentations, participants were provided with lunch and refreshments, followed by a question and answer session before the close. |

| | |
|---|---|
| | Promotional material was provided through the sales team at Henry Colbeck Ltd and owners/managers ordered the free case of packing using their usual method of ordering through Henry Colbeck Ltd. |
| 5. Who provided [page 8] | The educational material delivered by Henry Colbeck Ltd were developed by themselves in conversation with the research team. Additionally, Owner A, created his own presentation material and Owner B, presented without supporting material.<br><br>The goal setting, action planning and pledge form (behavioural contract) was developed by the research team, guided by VAS, health psychology expert. The research team provided all material to enable participants to record sales data in the required format. |
| 6. How [page 8] | Training delivered in person in a group session. |
| 7. Where [page 8] | Hotel, in close proximity to Henry Colbeck Ltd's office and warehouse facility, Gateshead. |
| 8. When and how much [page 8] | One session approximately three hours. |
| 9. Tailoring [page 8] | The material provided were tailored specifically to Fish & Chips Shops that offered a takeaway service. |
| 10. Modifications [page 8] | This was the first time the intervention had been delivered. However, previous material circulated by Henry Colbeck Ltd to its customers had been adapted and incorporated accordingly to the setting of delivering the information 'in-person'. |
| 11. Planning/fidelity (ways to maintain fidelity) [page 8] | Members of the research team attended the engagement event to assess if it was delivered as intended. As the event was a one-off no strategies were implemented to maintain or improve fidelity. |
| 12. Actual fidelity [page 8] | All presentations were delivered as intended. However, the goal setting activity was not. Participants had little time to complete the form and no support. During follow-up interviews many could not recall completing this or had not seen any value in the pledge. |

## References


1. Hoffmann TC, Glasziou PP, Boutron I, et al. Better reporting of interventions: template for intervention description and replication (TIDieR) checklist and guide. BMJ : British Medical Journal 2014;**348** doi: 10.1136/bmj.g1687[published Online First: Epub Date]|.

2. Jaworowska A, M. Blackham T, Long R, et al. Nutritional composition of takeaway food in the UK. Nutrition & Food Science 2014;**44**(5):414-30 doi: doi:10.1108/NFS-08-2013-0093[published Online First: Epub Date]|.

3. Goffe L, Rushton S, White M, et al. Relationship between mean daily energy intake and frequency of consumption of out-of-home meals in the UK National Diet and Nutrition Survey. International Journal of Behavioral Nutrition and Physical Activity 2017;**14**(1):131 doi: 10.1186/s12966-017-0589-5[published Online First: Epub Date]|.

4. Pereira MA, Kartashov AI, Ebbeling CB, et al. Fast-food habits, weight gain, and insulin resistance (the CARDIA study): 15-year prospective analysis. The Lancet 2005;**365**(9453):36-42 doi: http://dx.doi.org/10.1016/S0140-6736(04)17663-0[published Online First: Epub Date]|.

5. Bodicoat DH, Carter P, Comber A, et al. Is the number of fast-food outlets in the neighbourhood related to screen-detected type 2 diabetes mellitus and associated risk factors? Public Health Nutrition 2014;**FirstView**:1-8 doi: doi:10.1017/S1368980014002316[published Online First: Epub Date]|.

6. Hollands GJ, Shemilt I, Marteau TM, et al. Portion, package or tableware size for changing selection and consumption of food, alcohol and tobacco. Cochrane Database Syst Rev 2015;**9**:CD011045 doi: 10.1002/14651858.CD011045.pub2[published Online First: Epub Date]|.

7. Michie S, Richardson M, Johnston M, et al. The behavior change technique taxonomy (v1) of 93 hierarchically clustered techniques: building an international consensus for the reporting of behavior change interventions. Annals of behavioral medicine : a publication of the Society of Behavioral Medicine 2013;**46**(1):81-95 doi: 10.1007/s12160-013-9486-6[published Online First: Epub Date]|.

8. Adams J, Giles EL, McColl E, et al. Carrots, sticks and health behaviours: a framework for documenting the complexity of financial incentive interventions to change health behaviours. Health psychology review 2014;**8**(3):286-95 doi: 10.1080/17437199.2013.848410[published Online First: Epub Date]|.
