# Supplementary material for: Feasibility of working with a wholesale supplier to co-design and test acceptability of an intervention to promote smaller portions: an uncontrolled before-and-after study in British Fish & Chip shops
Source: BMJ Open. 2019 Feb 6;9(2):e023441. doi: 10.1136/bmjopen-2018-023441 (PMC6377521; doi:10.1136/bmjopen-2018-023441)
Supplement: Supplementary data [file bmjopen-2018-023441supp004.pdf]

Goffe et al. 2018. *Feasibility of working with a wholesale supplier to co-design and test acceptability of an intervention to promote smaller portions: an uncontrolled before-and-after study in British Fish & Chip Shops*

Customer survey

Interviewee ID	
Business name	
Date	

Fish and chip shop use

1. Do you use this fish and chip shop regularly?

Yes	No
☐	☐

2. What do you mean by regularly?

More than once a week	Once a week	Once every 2 weeks	Once a month	Once every 3 months	Once every 6 months	Once a year	First time
☐	☐	☐	☐	☐	☐	☐	☐

3. What are the main two reasons why you come to this fish and chip shop?

Taste/Quality	Convenience	Price	Portion size
☐	☐	☐	☐

Purchase behaviour

4. For how many people do you usually order for?

Write answer:

5. What do you normally order and what portion size? i.e. large/regular/smaller

Write answer:

Portion size

6. What do you think of the portion sizes here?

Too small	Just right	Too big
☐	☐	☐

Smaller portions

7. Are you aware of the smaller portions offered?

Yes	No
☐	☐

**8. Have you noticed the new posters or other forms of promotion in the shop?**	
Yes<br>☐	No<br>☐
**9. Have you tried the smaller portions?**	
Yes<br>☐	No<br>☐

**10. If not, would you buy it in future?**		
NA<br>☐	Yes<br>☐	No<br>☐

**11. If yes, who for?**
Write answer:
Other options
**12. Are there any meals/dishes/portion sizes you would like to see offered at this fish and chip shop?**
Write answer:

Gender	
Female<br>☐	Male<br>☐

Age category					
18 – 30<br>☐	31 - 40<br>☐	41 - 50<br>☐	51 - 60<br>☐	61 - 70<br>☐	Over 70<br>☐

Owner/manager interview topic guide

Interviewee background

- Please could you introduce yourself and state your role in the business
- Do you have any other premises?
- How long have you been working in fish and chips?
- How did you get into fish and chips?
- What is special or unique about your business?

Engagement with the project

- Did you attend the engagement event?
  - Why did you decide to attend the event?
  - What did you think about the event?
    - Prompts:
      - Speakers
      - Material
      - Location, i.e. convenience
      - Venue & refreshments
      - Henry Colbeck and their staff
      - Information delivered
  - What did you like?
  - Did you learn anything new?
  - Did you speak with other owners?
  - Any missing stakeholders?
  - Was there any part of the event you thought was missing, disliked or would change?
  - Was it worthwhile attending?
- If you didn't attend, did you speak with Henry Colbeck about portion control?
  - What did they tell you about portion control?
  - Was any of the information new?
  - What changes did they ask you to make?
  - What made you choose to give the posters/smaller portions a go?
  - Anything further at the time of the visit that could have been offered or suggested to support you to make changes?
- Did you complete a pledge sheet/Do you remember the pledge sheet?

Changes

- What changes have you made since attending the event?
  - Prompts
    - Increased awareness/regulation of portion control in your staff
    - Reduced portion sizes
    - Changed packaging
    - Introduced smaller portions
    - Put up poster(s)
- What's the role/importance of customer service?
- What response from customers?
  - Prompts
    - Changes in sales increase/decrease
    - Comments/Conversations
- Have you gained new customers?

Posters

- Where you offered both poster designs?
- Did you display them? And where? (possibly answered from previous questions)
  - If not, why not
  - If only inside/outside why?
- What did you think about the posters?
  - Prompts
    - Design
    - Wording
    - Appropriateness for your shop
- Any comments from customers? (possibly answered from previous questions)
- Could you suggest any changes?
- Will you keep the posters up?
- Did you do any further promotion?
  - Prompts
    - Staff led
    - Social media

Incentives

- What did you think of the incentives provided by Henry Colbeck?
  - Prompts
    - Loyalty scheme points
    - Free packaging
- Anything further you feel that they could have offered to support you in offering smaller portions/regulating portion size?

Sales data

- Did you record sales data?
- Was it easy to keep track of sales data?
    - Prompts
        - Lunchtime/Evening service
- Were the sales booklets that we provided of use?
- Have any of the changes you've made been financially beneficial?

Portion control

- Prior to this engagement with Henry Colbeck was portion control something that you considered?
- As a result of the engagement with Henry Colbeck do you give greater consideration to portion control?
- Is it important to you and your business to offer your customers a range of portion sizes?
- Will you continue to monitor the portion size of your meals?

Future plans

- Are you happy with the changes?
- Will you stick with the changes that you've made?
- Do you plan to make any further changes?

AOB

- Is there anything further you'd like to add about this process?

Intervention deliverer interview topic guide

Interviewee background

- Please could you introduce yourself and state your role in Henry Colbeck
- How long have you been working for Henry Colbeck?
- What do you feel separates Henry Colbeck from other suppliers in the industry?

Motivation for the work

- History of portion control
    - What work that you know of has been done broadly, what work has Henry Colbeck done specifically?
- Why were you interested in furthering such work (motives)?
    - What does your business stand to gain from such work?
    - Is it purely financial, or other broader enhancement of the industry?
- How did this specific work come to fruition?

Development

- What was your role in the development process?
- What was your role in the delivery process?
- How did you identify suitable project partners and speakers for the event?
- What role did the University research team play in the development of the intervention?
- Incentives, why were they chosen?
- Did it detract from your usual business operations?
- Was it costly to the business?

Engagement event

- Was it delivered as you had envisioned?
- What did you like most about the event?
- Any problems on the day?
- Any feedback from those that attended?
- Anything that you would do differently?

Posters

- Please could you detail the design process regarding the posters?
    - Prompts:
        - Costs
        - Time
        - Stakeholders
        - Designers
- You offered two posters, was there a preference for one over the other?
- Did you receive any feedback from owners?

- Anything learnt for future poster production?

Incentives

- You offered both loyalty scheme points and a free case of packaging, how were they received?
- How important did you feel they were to getting businesses to trial the smaller portions?
- Is there anything else that you considered offering?

Packaging

- You have a wide range of different packaging, was there a particular type you were interested in promoting as part of this work?
- Does the active promotion of specific packaging types raise any issues with customers, for examples those customers that have their own branded packaging?
- Did you receive any useful feedback from your customers?

Your assessment

- Overall how have your customers responded to this work?
- Do you feel that this project has been significant increase in your efforts to promote portion control and smaller portions compared to previous efforts?
- What is your overall assessment of the project? Are you happy? Was this extra and unnecessary work?
- What were the major learning issues for you?
- Did anything surprise you?
- Did you personally enjoy the work?
- Anything you didn't enjoy or found frustrating?
- Anything that you would do differently?
- What does Henry Colbeck, gain from this work?
- What have you gained personally from this work?

Future plans for smaller portions

- You have continued to the develop packaging for smaller portions, please can you detail what further work you done since the work with the feasibility study
- What was your motivation to continue on with this work?
- How receptive have your customers been?
- Do you have any other future work or ideas regarding portion control or reduced sized portions that you would like to detail?

AOB

- Is there anything further you'd like to add about this process?
