# Supplementary material for: Feasibility of working with a wholesale supplier to co-design and test acceptability of an intervention to promote smaller portions: an uncontrolled before-and-after study in British Fish & Chip shops
Source: BMJ Open. 2019 Feb 6;9(2):e023441. doi: 10.1136/bmjopen-2018-023441 (PMC6377521; doi:10.1136/bmjopen-2018-023441)
Supplement: Author's manuscript [file bmjopen-2018-023441.draft_revisions.pdf]

BMJ Open is committed to open peer review. As part of this commitment we make the peer review history of every article we publish publicly available.

When an article is published we post the peer reviewers' comments and the authors' responses online. We also post the versions of the paper that were used during peer review. These are the versions that the peer review comments apply to.

The versions of the paper that follow are the versions that were submitted during the peer review process. They are not the versions of record or the final published versions. They should not be cited or distributed as the published version of this manuscript.

BMJ Open is an open access journal and the full, final, typeset and author-corrected version of record of the manuscript is available on our site with no access controls, subscription charges or pay-per-view fees (http://bmjopen.bmj.com).

If you have any questions on BMJ Open's open peer review process please email info.bmjopen@bmj.com

BMJ Open

Feasibility of working with a wholesale supplier to co-design and test acceptability of an intervention to promote smaller portions in British Fish & Chip Shops

Journal:	*BMJ Open*
Manuscript ID	bmjopen-2018-023441
Article Type:	Research
Date Submitted by the Author:	27-Jun-2018
Complete List of Authors:	Goffe, Louis; Newcastle University, Institute of Health & Society; Newcastle University, Human Nutrition Research Centre<br>Hillier-Brown, Frances; Durham University, Faculty of Social Sciences and Health; Fuse – UKCRC Centre for Translational Research in Public Health<br>Hildred, Natalie; Fuse – UKCRC Centre for Translational Research in Public Health<br>Worsnop, Matthew; Henry Colbeck Limited<br>Adams, J; University of Cambridge, Centre for Diet & Activity Research<br>Araujo-Soares, Vera; Newcastle University, Institute of Health & Society; Fuse – UKCRC Centre for Translational Research in Public Health<br>Penn, Linda; Newcastle University, Institute of Health & Society; Fuse – UKCRC Centre for Translational Research in Public Health<br>Wrieden, Wendy; Newcastle University, Institute of Health & Society; Newcastle University, Human Nutrition Research Centre<br>Summerbell, Carolyn; Durham University, Faculty of Social Sciences and Health; Fuse – UKCRC Centre for Translational Research in Public Health<br>Lake, Amelia; Department of Science, School of Science, Engineering and Design, Teesside University; Fuse – UKCRC Centre for Translational Research in Public Health<br>White, Martin; University of Cambridge, Centre for Diet and Activity Research (CEDAR), MRC Epidemiology Unit; Newcastle University, Institute of Health & Society<br>Adamson, Ashley; Newcastle University, Institute of Health & Society; Fuse – UKCRC Centre for Translational Research in Public Health
Keywords:	Takeaways, Fish & Chips, food environments, Public health < INFECTIOUS DISEASES, diet, obesity

SCHOLARONE™
Manuscripts

Feasibility of working with a wholesale supplier to co-design and test acceptability of an intervention to promote smaller portions in British Fish & Chip Shops

Louis Goffe (LG louis.goffe@ncl.ac.uk M1.151 William Leech Building, Medical School, Newcastle University, Newcastle upon Tyne, NE2 4HH)[§1,2,3]

Frances Hillier-Brown (FHB frances.hillier-brown@durham.ac.uk)[4,3]

Natalie Hildred (NH)[3]

Matthew Worsnop (MWo)[5]

Jean Adams (JA jma79@medschl.cam.ac.uk)[6]

Vera Araujo-Soares (VAS vera.araujo-soares@ncl.ac.uk)[1,3]

Linda Penn (LP linda.penn@ncl.ac.uk)[1,3]

Wendy Wrieden (WW wendy.wrieden@newcastle.ac.uk)[1,2,3]

Carolyn D Summerbell (CDS carolyn.summerbell@durham.ac.uk)[4,3]

Amelia A Lake (AAL Amelia.Lake@tees.ac.uk)[7,3]

Martin White (MWh martin.white@mrc-epid.cam.ac.uk)[6,1]

Ashley Adamson (AA ashley.adamson@newcastle.ac.uk)[1,2,3]

[1] Institute of Health & Society, Newcastle University, Newcastle upon Tyne, UK

[2] Human Nutrition Research Centre, Newcastle University, Newcastle upon Tyne, UK

For peer review only

[3] Fuse – UKCRC Centre for Translational Research in Public Health, Newcastle upon Tyne, UK

[4] Faculty of Social Sciences and Health, Durham University, UK

[5] Newcastle Business School, Northumbria University, Newcastle upon Tyne; and Henry Colbeck Limited, Gateshead, UK

[6] Centre for Diet and Activity Research (CEDAR), MRC Epidemiology Unit, University of Cambridge, UK

[7] Department of Science, School of Science, Engineering and Design, Teesside University, UK

[§] Corresponding author

Word count: 3,817 (excluding tables)

For peer review only

For peer review only - http://bmjopen.bmj.com/site/about/guidelines.xhtml

ABSTRACT

Objectives

To explore the feasibility of working with a wholesale supplier to co-design and deliver, and to assess the acceptability of, an intervention to promote smaller portions in Fish & Chip Shops.

Design

Uncontrolled before-and-after study.

Setting

Fish & Chip Shops in northern England, 2016.

Participants

Owners (n=11), a manager, and customers (n=46) of Fish & Chip Shops; and intervention deliverers (n=3)

Intervention

Supplier-led, three-hour engagement event with shop owners and managers, highlighting the problem of excessive portion sizes and potential ways to reduce portion sizes; provision of box packaging to serve smaller portions; promotional posters and business incentives.

For peer review only

Data collection

In-store observations and sales data collected at baseline and post-intervention. Exit survey with customers. Semi-structured interviews with owners/managers and intervention deliverers post-intervention.

Results

Twelve Fish & Chip Shops were recruited. Observational data was collected from eight shops: at baseline, six shops did not promote the availability of smaller portion meals; at follow-up all eight did and five displayed the promotional poster. Seven out of 12 shops provided sales data and all reported increased sales of smaller portion meals post intervention. Of 46 customers surveyed: 28% were unaware of the availability of smaller portion meals; 20% had bought smaller portion meals; and 46% of those who had not bought these meals were interested to try them in the future. Interviews revealed: owners/managers found the intervention acceptable but wanted a clearer definition of a smaller portion meal; the supplier valued the experience of intervention co-production and saw the intervention as being compatible with their responsibility to drive innovation.

Conclusions

The co-design of the intervention with a supplier was feasible. The partnership facilitated the delivery of an intervention that was acceptable to owners and customers. Sales of smaller meal packaging suggest that promotion of such meals is viable and may be sustainable.

STRENGTHS AND LIMITATIONS OF THIS STUDY

- This is the first study we are aware of to evaluate the feasibility of working with a wholesale supplier to co-design and deliver a public health intervention targeting hot food takeaways.

For peer review only - http://bmjopen.bmj.com/site/about/guidelines.xhtml

- It is also the first study we are aware of to detail the potential role that wholesale suppliers can play in improving the healthfulness of food offerings from hot food takeaways, exemplified by Fish & Chip Shops.
- A mixed-method approach was employed, which successfully captured impacts of the interventions on all stakeholders.
- Data available on customer behaviours were limited and did not include takeaway food consumption.
- We had a small sample size, focused on one takeaway cuisine type and therefore the results may not be generalisable beyond the setting of Fish & Chip Shops.

KEYWORDS

Takeaways; Fish & Chips; food environments; public health; diet; obesity; intervention; behaviour change

For peer review only - http://bmjopen.bmj.com/site/about/guidelines.xhtml

INTRODUCTION

Takeaway and fast food meals, particularly from independent businesses, have been found to deliver excessive portion sizes,[1] driving high energy consumption,[2] which is a major public health concern.[3] Our previous work found that adults and children who ate takeaway meals at least weekly consumed 63-87kcal and 55-168kcal per day, respectively, more than those who consumed such meals rarely.[4] High takeaway meal consumption has been linked to weight gain [5] and diet-related diseases.[6]

In the UK, 'Fish & Chips' are culturally embedded,[7] with an estimated 10,500 Shops across the UK.[8] Typical meals from Fish & Chip Shops consist of white fish in a batter of flour and water, and chipped potatoes, both deep fried in fat.[9] Portion size is a key driver of the high energy, fat and salt content of these meals. One survey found that the median energy content of 64 Fish & Chip meals was 1,658kcal,[1] representing 79% of a woman's and 64% of a man's estimated average energy requirement.[10] This underpins the need to reduce portion sizes as a means to promote population health.[11, 12]

Wholesale suppliers have an influence on what food is offered by independent hot food takeaways;[13] most outlets use a limited number of suppliers.[14] Wholesale suppliers have a clear interest in the financial viability of the sector. Professionals with experience of delivering interventions in this setting have expressed a need for greater involvement of wholesale suppliers in intervention delivery.[15] To date, limited research has involved suppliers.[13]

For peer review only

The aim of this study was to explore the feasibility of working with a wholesale supplier to co-design and deliver an intervention to promote smaller portion meals in Fish & Chip Shops; and the acceptability of this intervention to shop owners/managers and their customers.

METHODS

Intervention co-design

We approached Henry Colbeck Limited (HC), an independent specialist wholesaler for Fish & Chip Shops, supplying over 2,500 shops across northern England and Scotland,[16] within a partnership of three independent suppliers that supplies over 6,000 shops across the UK.[8] We asked HC if they would work with us to co-design and lead on the delivery of an intervention to encourage Fish & Chip Shops to improve portion control of all meals and actively promote smaller portion meals. Members of the research team and HC staff set out their respective positions and terms of partnership that included: for HC – responsibility for intervention development and intervention delivery (including costs), as well as data sharing; for the research team – responsibility for study design and co-ordination of data collection, independence of analysis and right to publish findings.

We discussed findings of our previous studies on independent takeaways [4, 17-21] with HC, and in turn they shared their knowledge, detailing meal packaging options currently used: boxes, trays, and paper wrapping. It was established that large portion size meals existed across the sector, driven primarily by high competition and a desire to offer customers 'value-for-money'. We agreed the dual-focus of an intervention to facilitate and promote: better portion control through use of box packaging that restricts portion size (in particular in comparison with paper wrapping); and active promotion of smaller portion meals to customers. We agreed that HC should promote the intervention to Fish & Chip Shop owners/managers primarily on the potential financial benefits for

For peer review only

businesses of portion control and smaller portion sizes. The intervention was theorised in detail by the research team at the outset (Supplementary File A).

Intervention description

The research team supported HC to develop a three hour engagement event held in April 2016 at a hotel in Gateshead, North-East England. Fish & Chip Shop owners, managers, and their staff were invited to attend by HC. The programme included sessions delivered by HC and two Fish & Chip Shop owners, and a question and discussion session involving all attendees.

Owners/managers were encouraged to place a greater emphasis on portion control of all meals served through box packaging, and to actively promote smaller portion meals. The potential financial benefits of these strategies were stressed throughout. Participants were presented with a range (by size and material) of boxes available to support serving of smaller portion meals, but the choice of packaging product was left to the discretion of the owner/manager. An enhanced action-planning activity developed by the research team included a goal-setting form (Supplementary File B).[22] This included a 'public pledge', where Fish & Chip Shop owners/managers detailed what changes they would make, how and when these would be delivered, and how confident they were to deliver them. Owners/managers were encouraged to keep these written pledges. We completed a template for intervention description and replication (TIDieR) checklist [23] to fully describe the intervention (Supplementary File C).

Following the engagement event, owners/managers were offered two copies of one of two different A0 size posters promoting smaller portion meals to display in their shop (Figure 1). These were delivered to shops within 16 days of the event. HC suggested that one poster could be displayed in

For peer review only - http://bmjopen.bmj.com/site/about/guidelines.xhtml

the shop, and the other made visible to potential customers passing the shop. Additional incentives offered by HC were a free case (containing 100 units) of box packaging (for either regular or small portion meals - chosen by the owner/manager) and HC customer loyalty scheme points.

[INSERT FIGURE 1]

Figure 1. Promotional A0 size poster options

Additional intervention delivery was undertaken by HC sales staff who visited shops where the owner/manager had expressed an interest in attending the engagement event but had been unable to attend. In these meetings HC provided an overview of the information presented at the event, including details of changes suggested. They asked the owner/manager to complete the goal-setting form, offered the incentives and posters, and explained the recording of the requested sales data (see further details below).

Recruitment to the intervention

HC purposively selected shops in northern England to be invited to the engagement event with the aim of recruiting shops: located in a range of socio-economic settings; both within and outside major conurbations; and either known to be likely to engage or whose likelihood to engage was unknown. HC sent a postal invitation one month prior to the engagement event. This included a tailored message to the owner/manager with an invitation for them to attend along with a member of their staff. Owners or managers who did not respond were contacted by phone by HC to confirm their attendance.

For peer review only - http://bmjopen.bmj.com/site/about/guidelines.xhtml

Study design

We used an uncontrolled before-and-after study design to explore the feasibility of working with HC to co-design and deliver the intervention and its acceptability to Fish & Chip Shop owners/managers and their customers.

Data collection

Fish & Chip Shop owners/managers

Data collected about owners and managers who attended the engagement event or took part in one-to-one visits, which included: owner or manager status; number of Fish & Chip Shops owned (if owner); and whether they attended the engagement event with a member of their staff. We recorded details on each shop's: geographical location; local authority; location description (e.g. city centre, village high street); Indices of Multiple Deprivation (IMD) decile of the locale;[24] seating provision; and Food Standards Agency food hygiene rating.[25]

Goal-setting

A digital image was taken of all goal-setting forms completed either at the engagement event or during additional intervention delivery at the shop visits.

Covert observations

Covert data collection was completed in a subset of participating shops that were accessible to the research team within available resources, at three time points (baseline, two and six weeks post-intervention) by members of the research team posing as customers. Data collected included the visibility (including the display of the promotional posters) and availability of smaller portion meals for all customers. One regular size meal and one smaller portion meal (where available) was

For peer review only - http://bmjopen.bmj.com/site/about/guidelines.xhtml

purchased from each shop. The shop menu description of the smaller portion meal was recorded as was the packaging used and meal cost. The components (battered fish, chips) of meals were later weighed within two hours of purchasing.

Sales of Fish & Chip meals

Participating shops were provided with a booklet to record sales of regular and smaller portion meals from at least five days before to at least five days after first receiving the posters.

Customer survey

Following the completion of the covert data collection (six weeks post-intervention), a customer survey was conducted within participating shops. Interviews were conducted in-person after customers had ordered their food and while they waited for their meals to be served. Questions covered awareness of availability of smaller portion meals, views on meal portion size and purchasing behaviour including: frequency of and reasons for purchasing; whether they had tried smaller portion meal; and willingness to try smaller portion meals in the future. Data were also collected on the gender and age-category of respondents.

Semi-structured interviews

All shop owners/managers who had engaged in the intervention were invited to participate in a semi-structured interview, either in-person or by telephone, to explore their experience of the intervention. Further interviews were conducted with those responsible for development and delivery of the intervention at HC to explore their experiences of the intervention. Interviews with HC were conducted by a researcher not involved in intervention development and delivery.

For peer review only

Data analysis

Descriptive statistics were used to summarise quantitative data. Interviews were transcribed verbatim checked against the audio recordings for accuracy and then anonymised. Thematic framework analysis with constant comparison was used to identify themes.[26]

RESULTS

Recruitment and retention

Thirty-one Fish & Chip Shop owners or managers were invited to attend the engagement event. Of these, 15 (48%) stated an intention to attend, nine (29%) attended and three did not attend but took part in one-to-one sessions with HC staff. Thus, 12 took part in the evaluation (39%) (Figure 2).

[INSERT FIGURE 2]

Figure 2. Portion control intervention participation and data collection flowchart

Shop setting

Shops were spread across nine local authorities in northern England. Six of the 12 shops provided a sit-in restaurant service. Shops were located in areas across all IMD 2015 deciles.[24] The FSA hygiene rating of the shops was high, with ten receiving a maximum rating of five (Table 1).

Goal-setting

Eleven owners/managers completed the goal-setting form. All considered that they were already providing all of their customers with opportunities to purchase smaller portion meals in some form.

For peer review only

For peer review only - http://bmjopen.bmj.com/site/about/guidelines.xhtml

249 The principal change to usual business practices that could be inferred from the forms was a public

250 pledge to promote smaller portion meals, primarily through displaying posters (in five cases; Table 1).

251

For peer review only

For peer review only - http://bmjopen.bmj.com/site/about/guidelines.xhtml

Table 1. Summary of data collected

Shop	Summary	ID01	ID02	ID03	ID04	ID05	ID06	ID07	ID08	ID09	ID10	ID11	ID12
**Participant position**	Owner = 11 Manager = 1	Manager	Owner	Owner	Owner	Owner	Owner	Owner	Owner	Owner	Owner	Owner	Owner
**Number of total Fish & Chip Shops owned**	Own 1 = 10 Own > 1 = 2	1	1	7	1	1	1	1	1	1	2	1	1
**Attended the engagement event (number attendees)**	Yes = 9 No = 3	Yes (2)	Yes (2)	Yes (1)	Yes (2)	No	Yes (1)	No	Yes (1)	Yes (2)	No	Yes (1)	Yes (1)
**Completed goal-setting form**	Yes = 11 No = 1	Yes	Yes	Yes	Yes	Yes	Yes	Yes	Yes	Yes	No	Yes	Yes
**Public pledge**		"Smaller box"	"Smaller box/ Display poster"	"Smaller portion"	"Already doing"	"Advertising"	"Will promote smaller portion"	"Already using"	"Will display promotion posters"	"Bio box"*	Not completed	"Already doing"	"Display poster & use on social media to promote lite bite"
**Covert observations conducted**	Yes = 8 No = 4	Yes	No	No	Yes	Yes	Yes	Yes	Yes	Yes	Yes	No	No
**Semi-structured interview**	Yes = 9 No = 3	Yes	Yes	No	Yes	Yes	Yes	No	No	Yes	Yes	Yes	Yes
**Number of customers surveyed**	Shop n = 5 Customers n = 46	7	0	0	4	0	13	0	0	10	12	0	0
**Semi-structured interview**	Yes = 9 No = 3	Yes (person)	Yes (telephone)	No	Yes (person)	Yes (person)	Yes (person)	No	No	Yes (telephone)	Yes (person)	Yes (telephone)	Yes (telephone)
**Shop region**	North-East = 8 Yorkshire = 3 North-West = 1	North-East	North-East	Yorkshire and The Humber	North-East	North-East	North-East	North-East	North-East	Yorkshire and The Humber	North-East	North-West	Yorkshire and The Humber
**Shop**		Centre of a	Residential	Residential	Shopping	Shopping	High street in	High street in	High street in	Centre of a	High street in	City centre	High street in

For peer review only - http://bmjopen.bmj.com/site/about/guidelines.xhtml

**location description**		rural village	area of a market town	area of a city	centre in a residential area of a metropolitan borough	centre in a residential area of a metropolitan borough	a seaside town in a metropolitan borough	a seaside village in a metropolitan borough	a seaside town in a metropolitan borough	rural village	a seaside town in a metropolitan borough		a residential area of a metropolitan borough
**Sit-in restaurant**	Yes = 6<br>No = 6	No	No	Yes	No	No	Yes	No	Yes	Yes	Yes	Yes	No
**IMD decile (where 1 is most deprived 10% of LSOAs)**		9	4	7	1	3	8	5	8	9	7	3	5
**Shop FSA hygiene rating**	0 (low) = 0<br>1 = 0<br>2 = 0<br>3 = 1<br>4 = 1<br>5 (high) = 10	5	5	3	5	5	5	5	5	5	4	5	5

* Bio boxes constructed from biodegradable material extracted from sugar cane.

For peer review only

For peer review only - http://bmjopen.bmj.com/site/about/guidelines.xhtml

Covert observations

We collected observational data from eight shops (Table 2). At baseline, only two shops clearly displayed availability of smaller portion meals. During at least one of the post-intervention visits (two or six weeks), all eight shops displayed availability of smaller portion meals. At baseline, one shop had smaller portion meals on their main menu, two provided smaller portion meals on their lunchtime menu only, two on their children's menu only, and two had no smaller portion meals on any menu. Post-intervention, five of the eight shops actively promoted smaller portion meals using a poster facing inside the shop, and two also displayed a poster facing outside. Of the other three: one had actively promoted their own branded smaller portion meals throughout the evaluation but not displayed the poster provided by HC; another had introduced a smaller portion meal by the six-week follow-up; and one only had a smaller portion on the children's menu. All but one shop used box packaging at baseline and all shops served their meals in boxes at follow-up.

Between baseline and six-week follow-up, we observed a 24g increase in mean weight of battered fish, a 61g decrease in mean weight of chips, and a 37g decrease in mean total meal weight of regular meals. With regards to the smaller portion meals, we observed a 2g decrease in mean weight of battered fish, a 26g decrease in mean weight of chips, and a 27g decrease in mean total meal weight.

For peer review only - http://bmjopen.bmj.com/site/about/guidelines.xhtml

Table 2. Summary of covert observation data collected from each shop

		Post-intervention	
	**Baseline**	Two-weeks	Six-weeks
**Clearly displaying smaller portion meals available to all customers**	Yes = 2<br>No = 6	Yes = 7<br>No = 1	Yes = 6<br>No = 2
**Smaller portion meals available to buy**	Yes = 6<br>No = 2	Yes = 6<br>No = 2	Yes = 8<br>No = 0
**Active promotion of smaller meals**	NA	Yes = 5<br>No = 3	Yes = 4<br>No = 4
**Packaging used for regular meal**	Boxes = 7<br>Paper = 1	Boxes = 8	Boxes = 8
**Weight of regular meal (g): battered fish**	Mean = 265.1	Mean = 277.9	Mean = 289.3
**Weight of regular meal (g): chips**	Mean = 399.9	Mean =384.9	Mean = 339.1
**Weight of regular meal (g): total**	Mean = 665.0	Mean = 662.8	Mean = 628.4
**Packaging used for smaller meal**	Boxes = 6	Boxes = 6	Boxes = 8
**Weight of smaller meal (g): battered fish**	Mean = 175.7	Mean = 170.7	Mean = 174.0
**Weight of smaller meal (g): chips**	Mean = 273.0	Mean = 233.7	Mean = 247.4
**Weight of smaller meal (g): total**	Mean = 448.7	Mean = 404.3	Mean = 421.4

For peer review only

Sales of Fish & Chip meals

Seven shops returned usable sales data covering a mean of seven days pre- and 32 days post-delivery of the posters. Five used booklets provided by the study team and two used their own sheet to record sales data; only three split sales between lunch and evening periods. The mean proportion of all meals sold which were smaller portion was 14.2% pre-intervention and 21.2% post-intervention. One shop did not return sales data due to illness, the remaining three did not provide a reason.

Customer survey

Five owners/managers permitted customer surveys to be conducted in their shops (Table 1). A total of 46 questionnaires were completed (Table 3). Most customers surveyed bought meals once a month or more, choosing the shop for taste or convenience. Most were aware that smaller portion meals were available (72%) though only 20% had purchased them. Of those who had not previously tried smaller portion meals, 46% said they would be interested in trying them in the future.

For peer review only - http://bmjopen.bmj.com/site/about/guidelines.xhtml

Table 3. Customer survey responses

Variable	Level	n (%)
**Gender**	Female	21 (46)
	Male	25 (54)
**Age category**	18 – 30	10 (22)
	31 – 40	11 (24)
	41 – 50	2 (4)
	51 – 60	8 (17)
	61 – 70	7 (15)
	> 70	8 (17)
**Regular customer**	Yes	35 (76)
	No	11 (24)
**How regular**	More than once a week	2 (4)
	Once a week	10 (22)
	Once every 2 weeks	4 (9)
	Once a month	10 (22)
	Once every 3 months	6 (13)
	Once every 6 months	5 (11)
	Once a year	0 (0)
	First time	9 (20)
**Reasons for buying (up to 2 choices)**	Taste/Quality	29
	Convenience	32
	Price	3
	Portion size	0
	Other	1
**Portion sizes**	Too small	0 (0)
	Just right	36 (78)
	Too big	2 (4)
	NA (first time customer)	8 (17)
**Know about small portion**	Yes	33 (72)
	No	13 (28)
**Notice posters (where known to have been displayed)**	Yes	10 (37)
	No	17 (63)
**Tried the promoted smaller portion meals**	Yes	9 (20)
	No	37 (80)
**Try in the future**	Yes	17 (37)
	No	20 (43)
	NA (previously tried)	9 (20)

Semi-structured interviews

Interviews with owners/managers

Interviews were conducted with eight owners and one manager, five in person and four by telephone (Table 1). Thematic analysis identified six main themes.

For peer review only

For peer review only - http://bmjopen.bmj.com/site/about/guidelines.xhtml

Relationship between owner/manager and Henry Colbeck Limited

Those who had attended the engagement event were more enthusiastic about the process than those who received the intervention in a one-to-one session. They reported that the event provided a *"unique"* opportunity to speak about an industry matter with their peers. They were impressed with the speakers, but did not value the goal-setting form. Participants felt well-supported by HC throughout. While they appreciated the incentives offered by HC they did not feel these were necessary.

Suggested changes and smaller portion meal definitions

All respondents considered they were already providing smaller portion meals in some form at baseline. For most, therefore, it was the posters that constituted the intervention; whereas others reported developing a detailed strategy to promote smaller meal portions. One owner suggested industry-wide standards regarding portion sizes but acknowledged that *"universal adoption"* was unlikely.

Posters and shop setting

There was a mixed response to the posters. Some respondents felt they were 'fit-for-purpose', whereas others felt they did not fit with their shop's ethos. The only manager who attended the engagement event, stated that the shop's owner felt the poster design interfered with the shop's branding and therefore did not display it. Another owner suggested a preference for alternative promotional material (e.g. leaflet), detailing the *"benefits of buying smaller"*.

For peer review only - http://bmjopen.bmj.com/site/about/guidelines.xhtml

Importance of quality customer service

All respondents stressed the importance of high quality customer service to delivering smaller portion meals. One owner who had been involved in the Fish & Chip industry *"Pretty much all my life"*, had implemented numerous changes following the intervention with support from a shop owner who spoke at the engagement event. One owner who had been unable to attend the event, reported that they had not implemented smaller portion meals in the evening as they could not rely on serving staff to deliver these consistently.

Customer feedback

Few respondents reported receiving feedback on smaller portion meals from customers. However, one detailed the enthusiasm from a group of builders who had seen the posters and welcomed the change.

The ease of recording sales data

Those who provided sales data, reported that this had been straight forward. However, till systems used to monitor sales meant that, for most, it was not possible to distinguish between lunch and evening sales.

Interviews with those who developed and delivered the intervention

Interviews were conducted with three people from HC involved in intervention development and delivery. Thematic analysis identified five themes.

For peer review only - http://bmjopen.bmj.com/site/about/guidelines.xhtml

Intervention deliverers' motivations regarding intervention delivery

HC representatives viewed individual shops as focused on daily sales meaning it was up to HC to take *"strategic long-term views of the industry"*. Respondents wanted shops to succeed in an increasingly competitive marketplace. They took responsibility for driving sector innovation and acknowledged that *"I need Fish & Chip Shops to stay in business because they pay my wages"*.

Considerations in development of the intervention

HC representatives stated that effective engagement on smaller portion sizes with owners/managers could only be achieved by highlighting the financial and customer service benefits. HC staff saw themselves as providing information and choice to their customers, *"It is up to the customer [business owner] to make that choice, we are not going to force them to do anything"*. They were also happy to take the lead on intervention development and delivery and the cost to HC was viewed as an *"investment"*.

Intervention deliverers' views on acceptability

Representatives of HC were disappointed with attendance at the engagement event (9 of 31) and were frustrated that some shops *"didn't realise the potential"*. While many had not provided a reason for non-attendance, some reportedly told HC that it was due to staffing issues. However, the responses HC received from those who did attend was positive, *"I had quite a lot of people ring up and thanking me for the event"*, and they had viewed it as a rare *"interactive"* event: *"Fish fryers in the same room sharing ideas and you could see people writing down notes and bringing up their own problems. Other people were listening, engaging and offering advice and help. That doesn't happen often enough"*.

For peer review only - http://bmjopen.bmj.com/site/about/guidelines.xhtml

HC staff were aware that not all businesses would display the posters, with some owners/managers reporting that they were too big. However, they felt that it was important to provide the means to clearly distinguish between regular and smaller portion meals.

HC also viewed the incentive component of the intervention as an act of *"goodwill"* showing their commitment to the intervention. They did not see incentives as imperative to owner/manager involvement and, indeed, not all shops took advantage of them.

Future plans

As a direct result of this work, HC staff developed specific packaging for smaller portion meals and associated promotional material. At the time of interviews they were also trying to source smaller fish fillets for this new packaging.

While HC staff saw smaller portion packaging as a sustainable change, more sustainable methods of delivering the portion control message were required. The engagement event and one-to-one visits were not considered scalable or efficient, *"I can't go around and visit thousands of Fish & Chip Shops because I am only one person"*.

Experience of working with the research team

HC staff found working with the research team a positive and *"enjoyable experience"*. It had *"re-stimulated our [HC's] efforts"* and was felt to be rewarding for both parties.

For peer review only

For peer review only - http://bmjopen.bmj.com/site/about/guidelines.xhtml

DISCUSSION

Statement of principal findings

We found it was feasible to co-design and deliver an intervention to promote smaller portions with a commercial partner and the intervention was acceptable to both Fish & Chip Shops and their customers. Attendees at the engagement event valued and enjoyed it. Shop owners/managers were broadly willing to introduce and promote smaller portion meals although not all engaged with all aspects of the intervention. We successfully measured portions sizes and collected sales figures. We observed a reduction in both regular and smaller portion meals after the intervention and an increase in the proportion of meals sold that were a smaller portion. It was interesting to note that the reduction in portion size of regular meals was due to reduction in chips, that is, the least nutrient-dense component of the meal. The evaluation was conducted independently, avoiding the potential for competing interests of the commercial partners.

Strengths and limitations of the study

To our knowledge, this is the first study to evaluate the feasibility of working with a wholesale supplier to co-design and deliver a public health intervention, and also to demonstrate the potential role of wholesale suppliers in improving the food offerings from hot food takeaways.

Covert observations, while feasible here, would be resource intensive in a larger study. While participating shops were of a high standard, as measured by FSA hygiene rating,[25] they covered a wide range of IMD 2015 deciles,[24] which suggests that the intervention may be feasible across a range of socio-economic areas. Acceptability to shops with lower hygiene ratings may be lower. Goal-setting through the ‘public pledge’ was not found to be useful. Greater clarity concerning what was expected of shop owners/managers may have improved this. Some shops did not make use of

For peer review only

posters and other marketing materials may have engaged a wider range of shops. Some aspects of the intervention were not felt to be sustainable by the commercial partner and further thought is required to determine how any such intervention could be scaled-up. While we gained a good understanding of implementation within shops and impacts on sales, we had no measures of impact on customers' total diets or total population impact.

Strengths and limitations in relation to other studies

There are a limited number of intervention studies targeting takeaways in England, and few have been evaluated.[21] Most interventions to date have been delivered by local authorities, limiting their geographical reach. Suppliers, such as HC, have a much greater geographical reach. While mandatory approaches to portion control may be more effective than voluntary schemes,[27, 28] these would be harder to implement.[29] Our intervention is a rare voluntary, industry-led approach to portion control. Difficulties engaging independent takeaways in public health interventions have been previously described.[15] The 29% recruitment rate we achieved compares favourably to other interventions in the sector.[30]

Study implications

Our findings suggest that, within the takeaway sector, it is feasible to develop a supplier-led intervention based on 'providing information' and 'enabling choice'[31] and that this is acceptable across stakeholders. We highlight the importance of product specific packaging that constrains portion size, which can, in part, offset variability in servers' ability to deliver consistent portions. HC's resulting smaller portion box packaging was designed and branded to deliver a smaller sized 'Lite-BITE®' meal.[32] Sales of these boxes provide evidence of longer-term viability; in 2017 HC sold 5,523 cases (each of 100 units) of the 'Lite-BITE®' boxes to 253 unique accounts (personal communication). Takeaway owners/managers seem likely to be more receptive to messages about

For peer review only - http://bmjopen.bmj.com/site/about/guidelines.xhtml

portion control from peers than external organisations, framed primarily in the context of the potential financial benefits. The individual responsible for implementing changes in a takeaway (usually the owner or manager) may require clear and practical instructions on how to make suggested changes. Ideally, interventions should seek to engage with takeaway owners, or those responsible for branding, and this person should communicate changes to serving staff, within the wider context of good 'customer service'.

Unanswered questions and future research

Due to the costs, HC are not planning further engagement events as delivered in this intervention. An alternative platform to deliver the information and guidance in a collective format may be required to maximise the potential of smaller portion packaging (e.g. seminars at trade events). Future research could explore the impact of smaller portion meals in Fish & Chip Shops on customers' diet and the wider public health implications, as well as the potential to promote smaller meal portions through trade organisations and their events. While this intervention was feasible in a sample of Fish & Chip Shops, further work should seek to identify other sectors of the takeaway and wider catering industry where such an approach could be applied.

ETHICS

The study received ethical approval by the Newcastle University Research Ethics Committee. All Fish & Chip Shop owners/managers and those from HC who delivered the intervention provided written informed consent prior to participation. All customers who participated in the customer survey were provided with study information and provided oral consent prior to participation. Owners/managers were not required to give specific consent for the covert observation component of the data collection.

For peer review only

For peer review only - http://bmjopen.bmj.com/site/about/guidelines.xhtml

ACKNOWLEDGEMENTS

We thank Duncan McLean (director) and Bill Colbeck (chairman) of Henry Colbeck Limited for their time and commitment of resources to delivering the intervention, as well as their patience, understanding and respect of the study's research process. We would also like thank Laura Cutler, Eimear Duffy, Joel Halligan, Eimear Mullen and Maisie Rowland from the Human Nutrition Research Centre, Newcastle University who provided invaluable help with data collection.

FUNDING

This paper presents independent research funded by the National Institute for Health Research (NIHR) School for Public Health Research (SPHR). The views expressed in this publication are those of the author(s) and not necessarily those of the NHS, NIHR or the Department of Health. The SPHR was not involved in the design of the study, analysis, interpretation of data nor writing the manuscript.

This research was part of the SPHR funded project: *Transforming the 'foodscape': development and feasibility testing of interventions to promote healthier takeaway, pub or restaurant food,* with additional support from Durham and Newcastle Universities, SPHR is funded by the National Institute for Health Research (NIHR). At the time of funding SPHR was a partnership between the Universities of Sheffield, Bristol, Cambridge, Exeter, University College London; The London School for Hygiene and Tropical Medicine; the LiLaC collaboration between the Universities of Liverpool and Lancaster; and Fuse, the Centre for Translational Research in Public Health, a collaboration between Newcastle, Durham, Northumbria, Sunderland and Teesside Universities. Authors LG, FHB, NH, VAS, LP, CDS, AAL and AA are members of Fuse. Funding for Fuse comes from the British Heart

For peer review only

For peer review only - http://bmjopen.bmj.com/site/about/guidelines.xhtml

Foundation, Cancer Research UK, Economic and Social Research Council, Medical Research Council, the National Institute for Health Research, under the auspices of the UK Clinical Research Collaboration, and is gratefully acknowledged. AA is funded by the NIHR as a NIHR Research Professor. JA and MWh are funded by the Centre for Diet and Activity Research (CEDAR), a UKCRC Public Health Research Centre of Excellence. Funding from the British Heart Foundation, Cancer Research UK, Economic and Social Research Council, Medical Research Council, the National Institute for Health Research, and the Wellcome Trust, under the auspices of the UK Clinical Research Collaboration, is gratefully acknowledged.

COMPETING INTERESTS

The research funding contributed to the cost of design and production of some of the intervention materials. HC also contributed to the costs of intervention development and funded delivery. HC developed and sell the 'Lite-BITE®' Fish & Chip meal boxes detailed in the study. Neither the project, nor individual researchers, received financial contributions from HC for this study or any other work.

MWh is funded by NIHR as Director of its Public Health Research Funding Programme. AA is funded by NIHR as a Research Professor and the National Director of NIHR SPHR. CS and MWh are principal investigators in the NIHR SPHR. MWo led and delivered the intervention and provided the packaging materials; at the time of the research he was an undergraduate student at Newcastle Business School, Northumbria University on secondment to HC. After completion of the study (and his degree programme at Northumbria University), he became an employee of HC.

For peer review only - http://bmjopen.bmj.com/site/about/guidelines.xhtml

Henry Colbeck Limited

HC an independent supplier to over 2,500 Fish & Chip Shops in the North-East of England, Yorkshire, Cumbria and Scotland. HC is a private limited company founded in 1893 and based in Gateshead, North-East England. Company number 00822749 (www.colbeck.co.uk).

DATA SHARING STATEMENT

Anonymised data relating to the covert observations, sales of Fish & Chip meals, customer survey and semi-structured interviews are available on reasonable request from the corresponding author (LG).

AUTHOR CONTRIBUTIONS

JA, VAS, CDS, AAL, MWh & AA devised the concept for the Foodscape project. MWo developed the intervention with support from LG, FHB, VAS and AA. The evaluation study and associated methods were designed by LG, FHB, JA, VAS, LP, WW, CDS, MWh and AA. Data collection was overseen by LG and completed by LG, MWo, FHB, NH and AA. LG led on data analysis and drafting the manuscript, supported by MWh and AA. All authors provided critical comments on drafts of the manuscript, and read and approved the final version.

PATIENT INVOLVEMENT

No patients were involved in the study.

For peer review only

REFERENCES

1. Jaworowska A, M. Blackham T, Long R, et al. Nutritional composition of takeaway food in the UK. *Nutrition & Food Science* 2014;44(5):414-30. doi: doi:10.1108/NFS-08-2013-0093

2. Lachat C, Nago E, Verstraeten R, et al. Eating out of home and its association with dietary intake: a systematic review of the evidence. *Obesity Reviews* 2012;13(4):329-46. doi: 10.1111/j.1467-789X.2011.00953.x

3. Public Health England. Calorie reduction: The scope and ambition for action. In: Department of Health and Social Care, ed., 2018.

4. Goffe L, Rushton S, White M, et al. Relationship between mean daily energy intake and frequency of consumption of out-of-home meals in the UK National Diet and Nutrition Survey. *International Journal of Behavioral Nutrition and Physical Activity* 2017;14(1):131. doi: 10.1186/s12966-017-0589-5

5. Pereira MA, Kartashov AI, Ebbeling CB, et al. Fast-food habits, weight gain, and insulin resistance (the CARDIA study): 15-year prospective analysis. *The Lancet* 2005;365(9453):36-42. doi: http://dx.doi.org/10.1016/S0140-6736(04)17663-0

6. Bodicoat DH, Carter P, Comber A, et al. Is the number of fast-food outlets in the neighbourhood related to screen-detected type 2 diabetes mellitus and associated risk factors? *Public Health Nutrition* 2014;FirstView:1-8. doi: doi:10.1017/S1368980014002316

7. Walton JK. Fish and Chips, and the British Working Class, 1870-1940: A&C Black 1994.

8. Brooks J. Fish and Chips in Commercial Foodservice 2016: Seafish, 2017.

9. Seafish, AHDB Potatoes, Campden BRI. Enjoy Fish and Chips - Synopsis report. enjoyfishandchips.co.uk, 2016.

10. Scientific Advisory Committee on Nutrition. Dietary Reference Values for Energy: The Stattionery Office London, 2011.

For peer review only

For peer review only - http://bmjopen.bmj.com/site/about/guidelines.xhtml

11. Hollands GJ, Shemilt I, Marteau TM, et al. Portion, package or tableware size for changing selection and consumption of food, alcohol and tobacco. *Cochrane Database of Systematic Reviews* 2015(9) doi: 10.1002/14651858.CD011045.pub2

12. Marteau TM, Hollands GJ, Shemilt I, et al. Downsizing: policy options to reduce portion sizes to help tackle obesity. *BMJ* 2015;351 doi: 10.1136/bmj.h5863

13. Bagwell S, O'Keefe E, Doff S, et al. Encouraging healthier takeaways in low-income communities: Tools to support those working to encourage healthier catering amongst fast food takeaways. London: Cities Institute, London Metropolitan University, 2014.

14. Sharpe R. An inconvenient sandwich: the throwaway economics of takeaway food: New Economics Foundation 2010.

15. Goffe L, Penn L, Adams J, et al. The challenges of interventions to promote healthier food in independent takeaways in England: qualitative study of intervention deliverers' views. *BMC Public Health* 2018;18(1):184. doi: 10.1186/s12889-018-5096-3

16. Henry Colbeck Limited. Henry Colbeck Limited: About Us Gateshead2016 [Available from: http://www.colbeck.co.uk/about-us accessed Accessed October 2017.

17. Adams J, Goffe L, Brown T, et al. Frequency and socio-demographic correlates of eating meals out and take-away meals at home: cross-sectional analysis of the UK national diet and nutrition survey, waves 1-4 (2008-12). *International Journal of Behavioral Nutrition and Physical Activity* 2015;12(1):51.

18. Goffe L, Hillier-Brown F, Doherty A, et al. Comparison of sodium content of meals served by independent takeaways using standard versus reduced holed salt shakers: cross-sectional study. *International Journal of Behavioral Nutrition and Physical Activity* 2016;13(1):102. doi: 10.1186/s12966-016-0429-z

19. Goffe L, Wrieden W, Penn L, et al. Reducing the Salt Added to Takeaway Food: Within-Subjects Comparison of Salt Delivered by Five and 17 Holed Salt Shakers in Controlled Conditions. *PLOS ONE* 2016;11(9):e0163093. doi: 10.1371/journal.pone.0163093

For peer review only

For peer review only - http://bmjopen.bmj.com/site/about/guidelines.xhtml

20. Hillier-Brown FC, Summerbell CD, Moore HJ, et al. The impact of interventions to promote healthier ready-to-eat meals (to eat in, to take away or to be delivered) sold by specific food outlets open to the general public: a systematic review. *Obesity Reviews* 2016:n/a-n/a. doi: 10.1111/obr.12479

21. Hillier-Brown FC, Summerbell CD, Moore HJ, et al. A description of interventions promoting healthier ready-to-eat meals (to eat in, to take away, or to be delivered) sold by specific food outlets in England: a systematic mapping and evidence synthesis. *BMC Public Health* 2017;17(1):93. doi: 10.1186/s12889-016-3980-2

22. Michie S, Richardson M, Johnston M, et al. The behavior change technique taxonomy (v1) of 93 hierarchically clustered techniques: building an international consensus for the reporting of behavior change interventions. *Annals of behavioral medicine : a publication of the Society of Behavioral Medicine* 2013;46(1):81-95. doi: 10.1007/s12160-013-9486-6 [published Online First: 2013/03/21]

23. Hoffmann TC, Glasziou PP, Boutron I, et al. Better reporting of interventions: template for intervention description and replication (TIDieR) checklist and guide. *BMJ : British Medical Journal* 2014;348 doi: 10.1136/bmj.g1687

24. Department for Communities and Local Government. English indices of deprivation 2015. In: UK Government, ed., 2015.

25. Food Standards Agency. Food hygiene ratings, 2015.

26. Ritchie J, Lewis J, Nicholls CM, et al. Qualitative research practice: A guide for social science students and researchers: Sage 2013.

27. Lloyd-Williams F, Bromley H, Orton L, et al. Smorgasbord or symphony? Assessing public health nutrition policies across 30 European countries using a novel framework. *BMC Public Health* 2014;14(1):1195. doi: 10.1186/1471-2458-14-1195

For peer review only

602 28. McGill R, Anwar E, Orton L, et al. Are interventions to promote healthy eating equally effective for all? Systematic review of socioeconomic inequalities in impact. *BMC Public Health* 2015;15(1):457. doi: 10.1186/s12889-015-1781-7

605 29. Department of Health. Localising the Public Health Responsibility Deal: Toolkit for Local Authorities, 2013.

607 30. Hillier-Brown F, Lloyd S, Muhammad L, et al. Feasibility and acceptability of a Takeaway Masterclass aimed at improving healthier cooking practices and menu options in takeaway food outlets. *In Submission* 2018

610 31. Nuffield Council on Bioethics. Public health: ethical issues: Nuffield Council on Bioethics 2007.

611 32. Henry Colbeck Limited. The Lite-BITE® Gateshead2016 [Available from: http://www.colbeck.co.uk/sf-docs/resources/Lite-bitewebpage.pdf accessed Accessed March 2017.

For peer review only - http://bmjopen.bmj.com/site/about/guidelines.xhtml

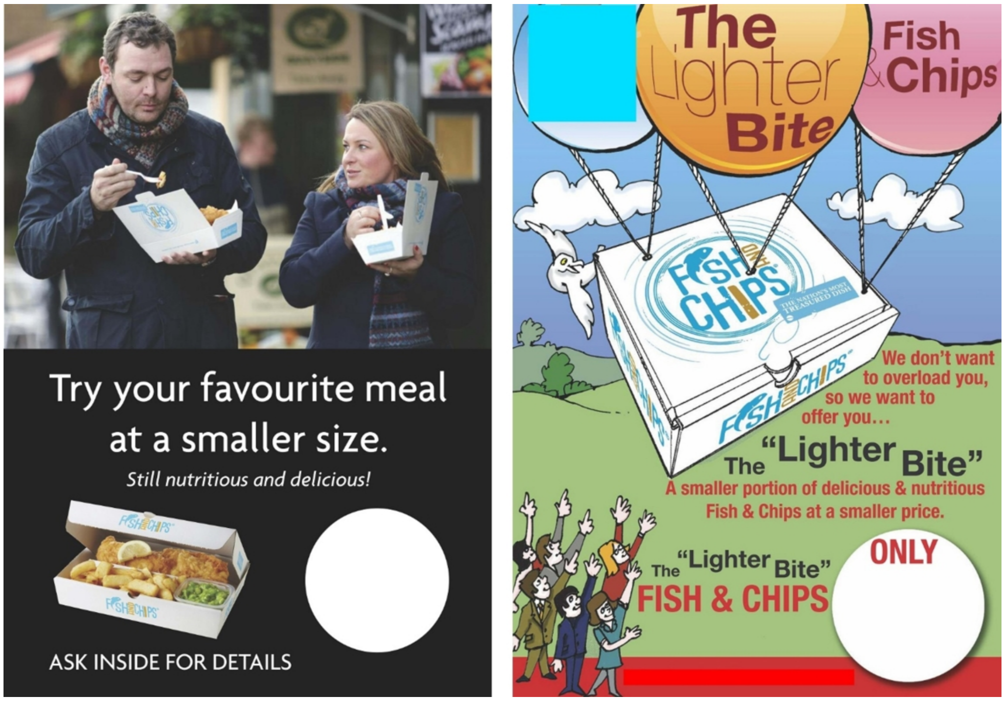

Figure 1. Promotional A0 size poster options

275x190mm (300 x 300 DPI)

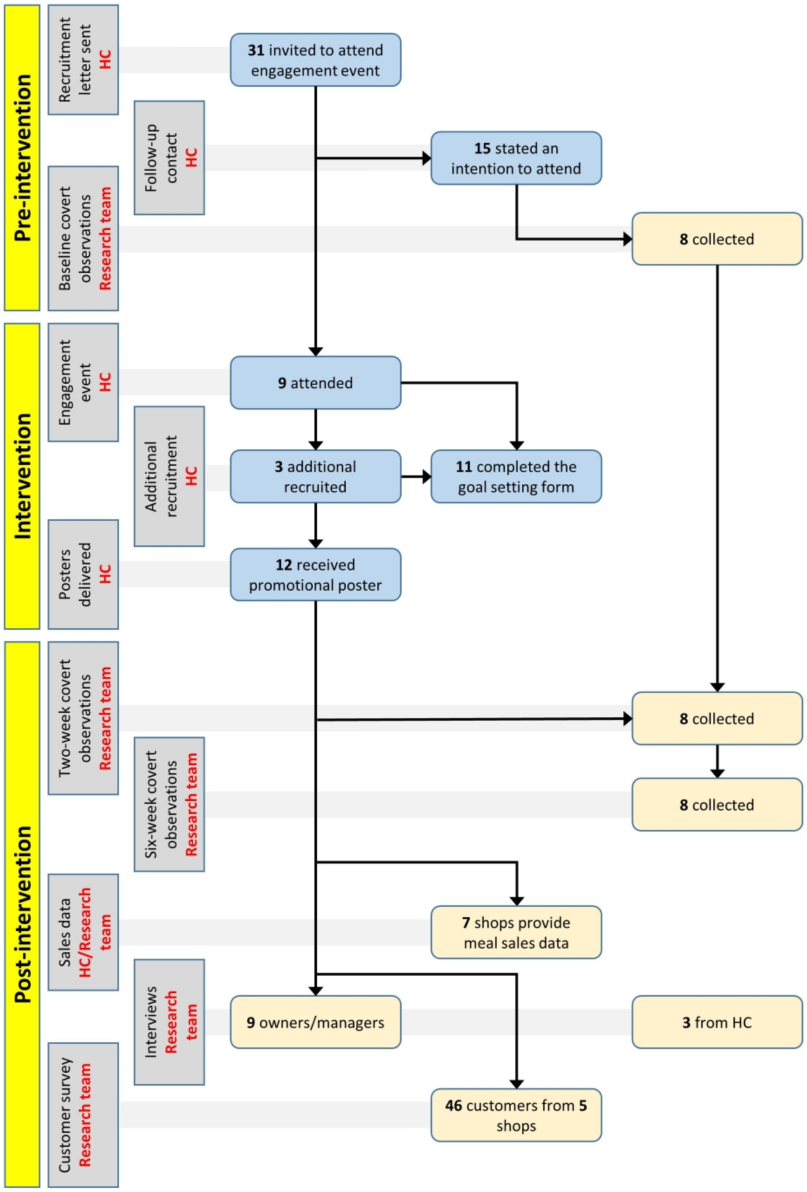

Figure 2. Portion control intervention participation and data collection flowchart

190x275mm (300 x 300 DPI)

Supplementary File A: Logic model

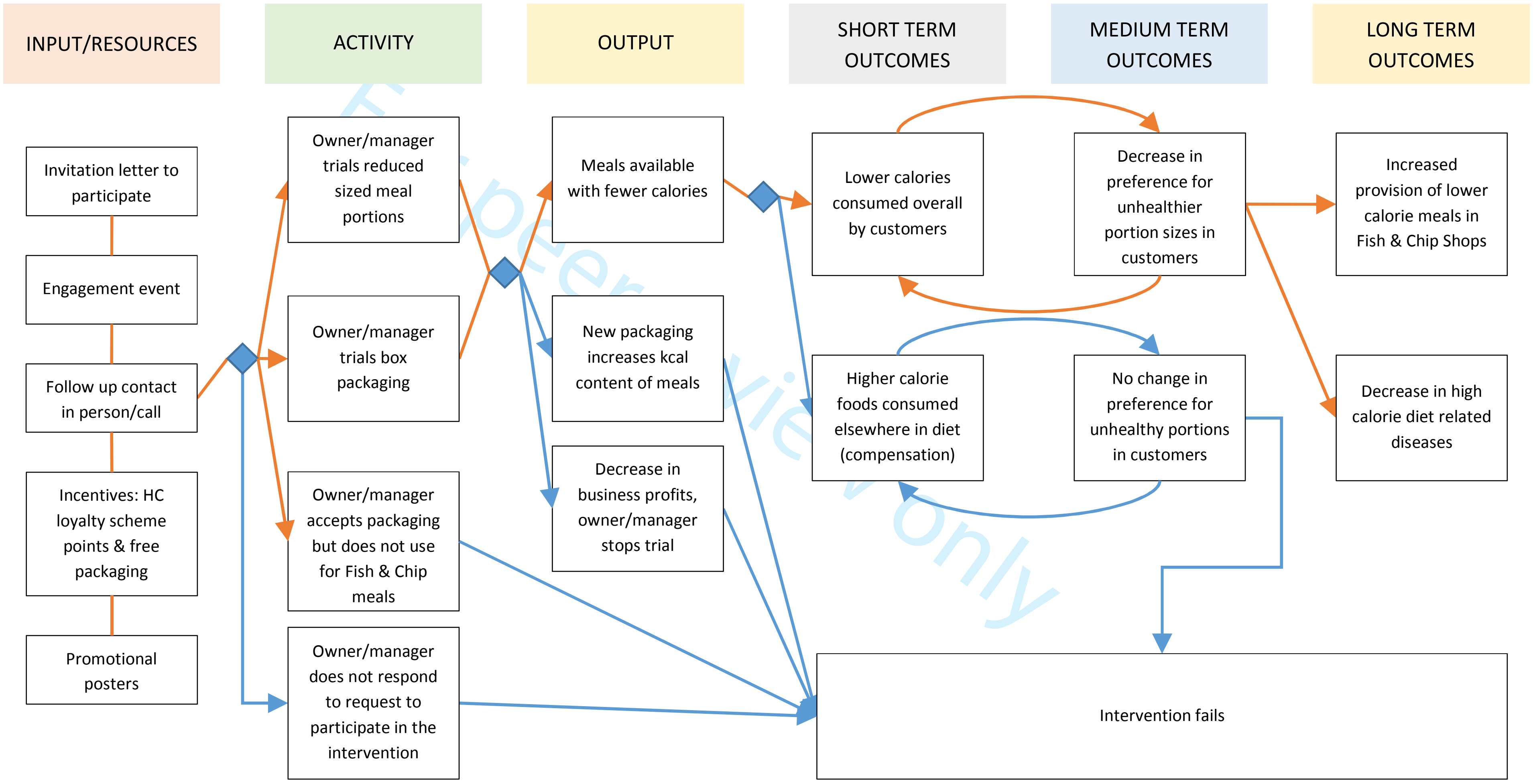

Supplementary File B: Goal-setting form

Team Spirit Event, Henry Colbeck Limited,

Wednesday 6th April 2016

Business name: ____

Business representatives:

Name: ____ Role: owner/ manager/ staff (circle answer)

Name: ____ Role: owner/ manager/ staff (circle answer)

From the topics covered at this Team Spirit event; what are the things you could change in your business?

	1. Could your business make this change? (Y/N)	2. Are you willing to try this change? (Y/N)
**Change to structured packaging:** *e.g. introduce bio boxes*		
**Make smaller portions available to your *customers* at *all times***		

**What to change?** *Structured packaging /Smaller portions*	**Which packaging will you use for which change?** *Single compartment corrugated cardboard box/Double compartment corrugated cardboard box/Bio box/MK*	**When will you do it?** *Date*	**How sure (confident) are you that your business can achieve this?** *From 1-5 (1=not at all sure; 5= very sure)**
1.			
2.			

Public Pledge: *Detail what, which and when you will change*

*1= not at all sure; 2= not very sure; 3= neither; 4= somewhat sure; 5= very sure

I give consent to be contacted both, by phone, or in person by Newcastle University to provide them with details of the:

- Packaging changes I have made (if any)
- The number of Fish & Chips portions (all sizes) sold before and after the project
- My comments and opinions of the Team Spirit event
- My comment and opinions of any changes I have made following the event

Additionally, I will allow Newcastle University to speak with a few of my customers to hear their thoughts.

Signed_______________________ Print Name__________________________

Phone Number (and best time to call) ______________________________

For peer review only - http://bmjopen.bmj.com/site/about/guidelines.xhtml

BMJ Open

Feasibility of working with a wholesale supplier to co-design and test acceptability of an intervention to promote smaller portions: an uncontrolled before-and-after study in British Fish & Chip Shops

Journal:	*BMJ Open*
Manuscript ID	bmjopen-2018-023441.R1
Article Type:	Research
Date Submitted by the Author:	26-Nov-2018
Complete List of Authors:	Goffe, Louis; Newcastle University, Institute of Health & Society; Newcastle University, Human Nutrition Research Centre<br>Hillier-Brown, Frances; Durham University, Department of Sport and Exercise Sciences; Fuse – UKCRC Centre for Translational Research in Public Health<br>Hildred, Natalie; Fuse – UKCRC Centre for Translational Research in Public Health<br>Worsnop, Matthew; Henry Colbeck Limited<br>Adams, J; University of Cambridge, Centre for Diet & Activity Research<br>Araujo-Soares, Vera; Newcastle University, Institute of Health & Society; Fuse – UKCRC Centre for Translational Research in Public Health<br>Penn, Linda; Newcastle University, Institute of Health & Society; Fuse – UKCRC Centre for Translational Research in Public Health<br>Wrieden, Wendy; Newcastle University, Institute of Health & Society; Newcastle University, Human Nutrition Research Centre<br>Summerbell, Carolyn; Durham University, Department of Sport and Exercise Sciences; Fuse – UKCRC Centre for Translational Research in Public Health<br>Lake, Amelia; Department of Science, School of Science, Engineering and Design, Teesside University; Fuse – UKCRC Centre for Translational Research in Public Health<br>White, Martin; University of Cambridge, Centre for Diet and Activity Research (CEDAR), MRC Epidemiology Unit; Newcastle University, Institute of Health & Society<br>Adamson, Ashley; Newcastle University, Institute of Health & Society; Fuse – UKCRC Centre for Translational Research in Public Health
**Primary Subject Heading**:	Public health
Secondary Subject Heading:	Nutrition and metabolism
Keywords:	Takeaways, Fish & Chips, food environments, Public health < INFECTIOUS DISEASES, diet, obesity

SCHOLARONE™
Manuscripts

Feasibility of working with a wholesale supplier to co-design and test acceptability of an intervention to promote smaller portions: an uncontrolled before-and-after study in British Fish & Chip Shops

Louis Goffe (LG louis.goffe@ncl.ac.uk M1.151 William Leech Building, Medical School, Newcastle University, Newcastle upon Tyne, NE2 4HH)[§1,2,3]

Frances Hillier-Brown (FHB frances.hillier-brown@durham.ac.uk)[4,3]

Natalie Hildred (NH)[3]

Matthew Worsnop (MWo)[5]

Jean Adams (JA jma79@medschl.cam.ac.uk)[6]

Vera Araujo-Soares (VAS vera.araujo-soares@ncl.ac.uk)[1,3]

Linda Penn (LP linda.penn@ncl.ac.uk)[1,3]

Wendy Wrieden (WW wendy.wrieden@newcastle.ac.uk)[1,2,3]

Carolyn D Summerbell (CDS carolyn.summerbell@durham.ac.uk)[4,3]

Amelia A Lake (AAL Amelia.Lake@tees.ac.uk)[7,3]

Martin White (MWh martin.white@mrc-epid.cam.ac.uk)[6,1]

Ashley Adamson (AA ashley.adamson@newcastle.ac.uk)[1,2,3]

[1] Institute of Health & Society, Newcastle University, Newcastle upon Tyne, UK

[2] Human Nutrition Research Centre, Newcastle University, Newcastle upon Tyne, UK

For peer review only

For peer review only - http://bmjopen.bmj.com/site/about/guidelines.xhtml

[3] Fuse – UKCRC Centre for Translational Research in Public Health, Newcastle upon Tyne, UK

[4] Department of Sport and Exercise Sciences, Durham University, Durham City, UK

[5] Newcastle Business School, Northumbria University, Newcastle upon Tyne; and Henry Colbeck Limited, Gateshead, UK

[6] Centre for Diet and Activity Research (CEDAR), MRC Epidemiology Unit, University of Cambridge, UK

[7] Department of Science, School of Science, Engineering and Design, Teesside University, UK

[§] Corresponding author

For peer review only

Word count: 4,013 (excluding tables)

For peer review only - http://bmjopen.bmj.com/site/about/guidelines.xhtml

ABSTRACT

Objectives

To explore the feasibility of working with a wholesale supplier to co-design and deliver, and to assess the acceptability of, an intervention to promote smaller portions in Fish & Chip Shops.

Design

Uncontrolled before-and-after study.

Setting

Fish & Chip Shops in northern England, 2016.

Participants

Owners (n=11), a manager, and customers (n=46) of Fish & Chip Shops; and intervention deliverers (n=3)

Intervention

Supplier-led, three-hour engagement event with shop owners and managers, highlighting the problem of excessive portion sizes and potential ways to reduce portion sizes; provision of box packaging to serve smaller portions; promotional posters and business incentives.

For peer review only

Data collection

In-store observations and sales data collected at baseline and post-intervention. Exit survey with customers. Semi-structured interviews with owners/managers and intervention deliverers post-intervention.

Results

Twelve Fish & Chip Shops were recruited. Observational data were collected from eight shops: at baseline, six shops did not promote the availability of smaller portion meals; at follow-up all eight did and five displayed the promotional poster. Seven out of 12 shops provided sales data and all reported increased sales of smaller portion meals post intervention. Of 46 customers surveyed: 28% were unaware of the availability of smaller portion meals; 20% had bought smaller portion meals; and 46% of those who had not bought these meals were interested to try them in the future. Interviews revealed: owners/managers found the intervention acceptable but wanted a clearer definition of a smaller portion meal; the supplier valued the experience of intervention co-production and saw the intervention as being compatible with their responsibility to drive innovation.

Conclusions

The co-design of the intervention with a supplier was feasible. The partnership facilitated the delivery of an intervention that was acceptable to owners and customers. Sales of smaller meal packaging suggest that promotion of such meals is viable and may be sustainable.

STRENGTHS AND LIMITATIONS OF THIS STUDY

- This is the first study we are aware of to evaluate the feasibility of working with a wholesale supplier to co-design and deliver a public health intervention targeting hot food takeaways.

For peer review only

- It is also the first study we are aware of to detail the potential role that wholesale suppliers can play in improving the healthfulness of food offerings from hot food takeaways, exemplified by Fish & Chip Shops.
- A mixed-method approach was employed, which successfully captured impacts of the interventions on all stakeholders.
- Data available on customer behaviours were limited and did not include takeaway food consumption.
- We had a small sample size, focused on one takeaway cuisine type and therefore the results may not be generalisable beyond the setting of Fish & Chip Shops.

KEYWORDS

Takeaways; Fish & Chips; food environments; public health; diet; obesity; intervention; behaviour change

For peer review only

INTRODUCTION

Takeaway and fast food meals, particularly from independent businesses, have been found to deliver excessive energy by means of large portion sizes,[1] driving high energy consumption,[2] which is a major public health concern.[3] Our previous work found that adults and children who ate takeaway meals at least weekly consumed 63-87kcal and 55-168kcal per day, respectively, more than those who consumed such meals rarely.[4] High takeaway meal consumption has been linked to weight gain [5] and diet-related diseases.[6]

In the UK, 'Fish & Chips' are culturally embedded,[7] with an estimated 10,500 shops nationally.[8] Typical Fish & Chip Shop meals consist of white fish in batter and chipped potatoes, both deep fried.[9] One survey found that the median energy content of 64 Fish & Chip meals was 1,658kcal,[1] representing 79% of a woman's and 64% of a man's estimated average daily energy requirement.[10] This suggests reducing portion sizes could be a means to promote population health.[11-14]

As most outlets use a limited number of wholesale suppliers,[15] these have substantial influence on what food is offered by independent hot food takeaways.[16] Whilst working with suppliers provides an opportunity for intervention,[17] to date, limited research has done so.[16]

The aim of this study was to explore the feasibility of working with a wholesale supplier to co-design and deliver an intervention to promote smaller portion meals in Fish & Chip Shops in northern England; and the acceptability of this intervention to shop owners/managers and their customers. It was not a process or outcome evaluation study.

For peer review only

METHODS

Intervention co-design

We approached Henry Colbeck Limited (HC), an independent specialist Fish & Chip Shops wholesaler, supplying over 2,500 shops across northern England and Scotland,[18] within a partnership supplying over 6,000 shops across the UK.[8] We asked HC if they would work with us to co-design and lead delivery of an intervention to encourage Fish & Chip Shops to improve portion control and promote smaller portion meals. Members of the research team and HC staff set out their respective positions and terms of partnership that included: for HC – responsibility for intervention development and intervention delivery (including costs), as well as data sharing; for the research team – responsibility for study design and co-ordination of data collection, independence of analysis and right to publish findings.

We discussed findings of our previous studies on independent takeaways [4, 19-23] with HC, and in turn they shared their knowledge, detailing meal packaging options currently used: boxes, trays, and paper wrapping. It was established that large portion size meals existed across the sector, driven primarily by high competition and a desire to offer customers 'value-for-money'. We agreed the dual-focus of an intervention to facilitate and promote: better portion control, supported through use of box packaging that standardises portion size (in particular in comparison with paper wrapping); and active promotion of smaller portion meals. We agreed that implementation of the intervention should not incur direct costs to the participating Fish & Chip Shops and that HC should promote to owners/managers primarily on the potential financial benefits of portion control and smaller portion sizes. HC recruited two owners with established smaller portion meal promotion to support intervention delivery to detail their practical experience and financial benefits. The intervention was theorised in detail by the research team (Supplementary File A).

For peer review only

For peer review only - http://bmjopen.bmj.com/site/about/guidelines.xhtml

Intervention description

The research team supported HC to develop a three hour engagement event held in April 2016 at a hotel in North-East England. Fish & Chip Shop owners, managers, and their staff were invited to attend by HC. The programme included sessions delivered by HC and two Fish & Chip Shop owners, followed by a question and discussion session.

Owners/managers were encouraged to place a greater emphasis on portion control by using box packaging, and to actively promote smaller portion meals. The potential financial benefits of attracting a wider customer base and reducing portion sizes without pro-rata reductions in price charged were stressed throughout, such as an increase in trade and higher meal profit margin. Participants were presented with a range (by size and material) of smaller portion boxes, but the choice of packaging selected was made by the owner/manager. An enhanced action-planning activity developed by the research team included a goal-setting form (Supplementary File B).[24] This included a 'public pledge', where Fish & Chip Shop owners/managers detailed what changes they would make, how and when these would be delivered, and how confident they were to deliver them. Owners/managers were encouraged to keep these pledges. We completed a template for intervention description and replication (TIDieR) checklist [25] (Supplementary File C).

Following the engagement event, owners/managers were offered two copies of one of two different A0 size posters promoting smaller portion meals for their shops (Figure 1). These were delivered to shops within 16 days. HC suggested that one poster could be displayed in-store, and the other made visible to passers-by. Additional incentives offered by HC were 100 units of the box packaging chosen by the owner/manager and HC customer loyalty scheme points.

For peer review only

[INSERT FIGURE 1]

Figure 1. Promotional A0 size poster options

Additional intervention delivery was undertaken by HC sales staff who visited owners/managers who had expressed an interest in the engagement event but had not attended. An overview of the information presented at the event was provided to owners/managers and they were asked to complete the goal-setting form, offered the incentives and posters, and the recording of sales data was explained (see below).

Recruitment to the intervention

HC purposively selected shops in northern England to be invited to the engagement event with the aim of recruiting shops: located in a range of socio-economic settings; both within and outside major conurbations; and either known to be likely to engage or whose likelihood to engage was unknown. HC sent a postal invitation one month prior to the engagement event. This included a tailored message to the owner/manager with an invitation for them to attend along with a member of their staff. Owners or managers who did not respond were contacted by phone by HC.

Study design

We used an uncontrolled before-and-after study design to explore the feasibility of working with HC to co-design and deliver the intervention and its acceptability to Fish & Chip Shop owners/managers and their customers.

For peer review only - http://bmjopen.bmj.com/site/about/guidelines.xhtml

Data collection

Fish & Chip Shop owners/managers

Data collected about owners/managers who attended the engagement event or took part in one-to-one visits included: owner or manager status; number of Fish & Chip Shops owned (if owner); and whether they attended the engagement event with a member of their staff. We recorded details on each shop's: geographical location; local authority; location description (e.g. city centre, village high street); Indices of Multiple Deprivation (IMD) decile of location;[26] seating provision; and Food Standards Agency food hygiene rating.[27]

Goal-setting

A digital image was taken of all completed goal-setting forms, to record pledged changes.

Covert observations

To provide objective measures of change, covert data collection was completed in a subset of participating shops that were accessible to the research team, at three time points (baseline, two and six weeks post-intervention). Members of the research team posed as customers. Data collected included the visibility (including the display of promotional posters) and availability of smaller portion meals. One regular size meal and one smaller portion meal (where available) was purchased from each shop. The shop menu description of the smaller portion meal was recorded as was the packaging used and meal cost. The components (battered fish, chips) of meals were weighed within two hours of purchasing.

For peer review only

Sales of Fish & Chip meals

Participating shops were provided with a booklet to record sales of regular and smaller portion meals from at least five days before to at least five days after first receiving the posters.

Customer survey

Following the completion of the six-week post-intervention covert data collection, a customer survey was conducted in participating shops (Supplementary File D). Interviews were conducted in-person after customers had ordered their food but before food had been served. Questions covered customer gender, age-group, awareness of availability of smaller portion meals, views on meal portion size and purchasing behaviour including: frequency of and reasons for purchasing; whether they had tried smaller portion meal; and willingness to try smaller portion meals in the future.

Semi-structured interviews

All shop owners/managers who engaged in the intervention were invited to participate in a semi-structured interview, either in-person or by telephone, to explore their experience of the intervention (Supplementary File D), conducted by LG. Interviews were also conducted with those responsible for development and delivery of the intervention at HC to explore their experiences of the intervention (Supplementary File D). Interviews with HC were conducted by a researcher, FHB, not involved in intervention development and delivery.

Data analysis

Descriptive statistics (sample size, means, proportions), conducted in R (LG), were used to summarise quantitative data but not for the purpose of statistical inference.[28] Interviews were transcribed verbatim checked for accuracy and then anonymised. Thematic framework analysis with constant

For peer review only

comparison was used to identify themes related to feasibility and acceptability of the intervention.[29] The coding framework for each set of interviews was based on a priori themes from the interview topic guides and emergent themes from the data. The final coding framework was then applied to all transcripts and the resulting themes were reviewed and agreed upon by team members involved in the analysis (LG, AA & MWh).

RESULTS

Recruitment and retention

Thirty-one Fish & Chip Shop owners or managers were invited to attend the engagement event. Of these, 15 (48%) stated an intention to attend, nine (29%) attended and three did not attend but took part in one-to-one sessions with HC staff. Thus, 12 took part in the evaluation (39%) (Figure 2).

[INSERT FIGURE 2]

Figure 2. Portion control intervention participation and data collection flowchart

Shop setting

Shops were spread across nine local authorities. Six of the 12 shops provided seating. Shops were located in areas across all IMD deciles.[26] The FSA hygiene rating of the shops was high, with ten receiving a maximum rating of five (Table 1).

Goal-setting

Eleven owners/managers completed the goal-setting form. All considered that they were already providing all of their customers with opportunities to purchase smaller portion meals in some form.

For peer review only - http://bmjopen.bmj.com/site/about/guidelines.xhtml

249 The principal change to usual business practices that could be inferred from the forms was a public
250 pledge to promote smaller portion meals, primarily through displaying posters (n=5; Table 1).

251

For peer review only

For peer review only - http://bmjopen.bmj.com/site/about/guidelines.xhtml

Table 1. Summary of data collected

	Shop-level summary (count)	Individual Fish & Chip Shop											
		ID01	ID02	ID03	ID04	ID05	ID06	ID07	ID08	ID09	ID10	ID11	ID12
**Participant position**	Owner = 11<br>Manager = 1	Manager	Owner	Owner	Owner	Owner	Owner	Owner	Owner	Owner	Owner	Owner	Owner
**Number of total Fish & Chip Shops owned**	Own 1 = 10<br>Own > 1 = 2	1	1	7	1	1	1	1	1	1	2	1	1
**Attended the engagement event (number attendees)**	Yes = 9<br>No = 3	Yes (2)	Yes (2)	Yes (1)	Yes (2)	No	Yes (1)	No	Yes (1)	Yes (2)	No	Yes (1)	Yes (1)
**Completed goal-setting form**	Yes = 11<br>No = 1	Yes	Yes	Yes	Yes	Yes	Yes	Yes	Yes	Yes	No	Yes	Yes
**Public pledge**		"Smaller box"	"Smaller box/ Display poster"	"Smaller portion"	"Already doing"	"Advertising"	"Will promote smaller portion"	"Already using"	"Will display promotion posters"	"Bio box"*	Not completed	"Already doing"	"Display poster & use on social media to promote lite bite"
**Covert observations conducted**	Yes = 8<br>No = 4	Yes	No	No	Yes	Yes	Yes	Yes	Yes	Yes	Yes	No	No
**Semi-structured interview**	Yes = 9<br>No = 3	Yes	Yes	No	Yes	Yes	Yes	No	No	Yes	Yes	Yes	Yes
**Number of customers surveyed**	Shop<br>n = 5<br>Customers<br>n = 46	7	0	0	4	0	13	0	0	10	12	0	0
**Semi-structured interview**	Yes = 9<br>No = 3	Yes (person)	Yes (telephone)	No	Yes (person)	Yes (person)	Yes (person)	No	No	Yes (telephone)	Yes (person)	Yes (telephone)	Yes (telephone)
**Shop region**	North-East = 8<br>Yorkshire = 3	North-East	North-East	Yorkshire and The Humber	North-East	North-East	North-East	North-East	North-East	Yorkshire and The Humber	North-East	North-West	Yorkshire and The Humber

For peer review only

	North-West = 1												
**Shop location description**		Centre of a rural village	Residential area of a market town	Residential area of a city	Shopping centre in a residential area of a metropolitan borough	Shopping centre in a residential area of a metropolitan borough	High street in a seaside town in a metropolitan borough	High street in a seaside village in a metropolitan borough	High street in a seaside town in a metropolitan borough	Centre of a rural village	High street in a seaside town in a metropolitan borough	City centre	High street in a residential area of a metropolitan borough
**Sit-in restaurant**	Yes = 6<br>No = 6	No	No	Yes	No	No	Yes	No	Yes	Yes	Yes	Yes	No
**IMD decile (where 1 is most deprived 10% of LSOAs)**		9	4	7	1	3	8	5	8	9	7	3	5
**Shop FSA hygiene rating (0 to 5, where 0 is low and 5 is high)**		5	5	3	5	5	5	5	5	5	4	5	5

* Bio boxes constructed from biodegradable material extracted from sugar cane.

For peer review only

Covert observations

We collected observational data from eight shops (Table 2). At baseline, only two shops clearly displayed availability of smaller portion meals. During at least one of the post-intervention visits (two or six weeks), all eight shops displayed availability of smaller portion meals. At baseline, one shop had smaller portion meals on their main menu, two provided smaller portion meals on their lunchtime menu only, two on their children's menu only, and two had no smaller portion meals on any menu. Post-intervention, five of the eight shops actively promoted smaller portion meals using an in-store facing poster, and two also displayed a poster facing outside. Of the other three: one actively promoted their own branded smaller portion meals throughout but did not displayed the HC posters; another had introduced a smaller portion meal by the six-week follow-up; and one only had a smaller portion on the children's menu. All but one shop used box packaging at baseline and all did so at follow-up.

Between baseline and six-week follow-up, we observed a 24g increase in mean weight of battered fish, a 61g decrease in mean weight of chips, and a 37g decrease in mean total meal weight of regular meals. With regards to the smaller portion meals, we observed a 2g decrease in mean weight of battered fish, a 26g decrease in mean weight of chips, and a 27g decrease in mean total meal weight.

For peer review only

For peer review only - http://bmjopen.bmj.com/site/about/guidelines.xhtml

Table 2. Summary of covert observation data collected from each shop

		Post-intervention	
	**Baseline**	Two-weeks	Six-weeks
**Clearly displaying smaller portion meals available to all customers**	Yes = 2<br>No = 6	Yes = 7<br>No = 1	Yes = 6<br>No = 2
**Smaller portion meals available to buy**	Yes = 6<br>No = 2	Yes = 6<br>No = 2	Yes = 8<br>No = 0
**Active promotion of smaller portion meals**	NA	Yes = 5<br>No = 3	Yes = 4<br>No = 4
**Packaging used for regular meal**	Boxes = 7<br>Paper = 1	Boxes = 8	Boxes = 8
**Weight of regular meal (g): battered fish**	Mean = 265.1	Mean = 277.9	Mean = 289.3
**Weight of regular meal (g): chips**	Mean = 399.9	Mean =384.9	Mean = 339.1
**Weight of regular meal (g): total**	Mean = 665.0	Mean = 662.8	Mean = 628.4
**Packaging used for smaller portion meal**	Boxes = 6	Boxes = 6	Boxes = 8
**Weight of smaller portion meal (g): battered fish**	Mean = 175.7	Mean = 170.7	Mean = 174.0
**Weight of smaller portion meal (g): chips**	Mean = 273.0	Mean = 233.7	Mean = 247.4
**Weight of smaller portion meal (g): total**	Mean = 448.7	Mean = 404.3	Mean = 421.4
**Cost of regular meal (£)**	Mean = £5.80	Mean = £5.79	Mean = £5.79
**Cost of smaller portion meal (£)**	Mean = £4.22	Mean = £4.07	Mean = £4.00

Sales of Fish & Chip meals

Seven shops returned usable sales data covering a mean of seven days pre- and 32 days post-delivery of the posters. However, this was inconsistent in format and detailed analyses were not possible. The mean proportion of all meals sold which were smaller portion was 14.2% pre-intervention and 21.2% post-intervention. One shop did not return sales data due to illness, the remaining three did not provide a reason.

Customer survey

Five owners/managers permitted customer surveys to be conducted in their shops (Table 1). A total of 46 questionnaires were completed (Table 3). Most customers surveyed bought meals once a month or more, choosing the shop for taste or convenience. Most were aware that smaller portion meals were available (72%) though only 20% had purchased them. Of those who had not previously tried smaller portion meals, 46% said they would be interested in trying them in the future.

For peer review only

For peer review only - http://bmjopen.bmj.com/site/about/guidelines.xhtml

Table 3. Customer survey responses

Variable	Level	n (%)
**Gender**	Female	21 (46)
	Male	25 (54)
**Age category**	18 – 30	10 (22)
	31 – 40	11 (24)
	41 – 50	2 (4)
	51 – 60	8 (17)
	61 – 70	7 (15)
	> 70	8 (17)
**Regular customer**	Yes	35 (76)
	No	11 (24)
**How regular**	More than once a week	2 (4)
	Once a week	10 (22)
	Once every 2 weeks	4 (9)
	Once a month	10 (22)
	Once every 3 months	6 (13)
	Once every 6 months	5 (11)
	Once a year	0 (0)
	First time	9 (20)
**Reasons for buying (up to 2 choices)**	Taste/Quality	29
	Convenience	32
	Price	3
	Portion size	0
	Other	1
**Portion sizes**	Too small	0 (0)
	Just right	36 (78)
	Too big	2 (4)
	NA (first time customer)	8 (17)
**Know about small portion**	Yes	33 (72)
	No	13 (28)
**Notice posters (where known to have been displayed)**	Yes	10 (37)
	No	17 (63)
**Tried the promoted smaller portion meals**	Yes	9 (20)
	No	37 (80)
**Try in the future**	Yes	17 (37)
	No	20 (43)
	NA (previously tried)	9 (20)

Semi-structured interviews

Interviews with owners/managers

Interviews were conducted with eight owners and one manager, five in person and four by telephone (Table 1). Thematic analysis identified six main themes.

For peer review only - http://bmjopen.bmj.com/site/about/guidelines.xhtml

Relationship between owner/manager and Henry Colbeck Limited

Those who had attended the engagement event were more enthusiastic about the process than those who received the intervention in a one-to-one session. They reported that the event provided a *"unique"* [ID06, ID04] opportunity to speak about an industry matter with their peers. They were impressed with the speakers, but did not value the goal-setting form. Participants felt well-supported by HC throughout. While they appreciated the incentives offered by HC they did not feel these were necessary.

Suggested changes and smaller portion meal definitions

All respondents considered they were already providing smaller portion meals in some form at baseline. For most, therefore, the intervention constituted the posters; whereas others reported developing a detailed strategy to promote smaller meal portions. One owner suggested industry-wide standards regarding portion sizes but acknowledged that *"universal adoption"* [ID12] was unlikely.

Posters and shop setting

There was a mixed response to the posters. Some respondents felt they were *"really good… it really just says it all… it's [poster] very relevant to our business"* [ID05], whereas others felt they did not fit with their shop's ethos. The only manager who attended the engagement event, stated that the shop's owner felt the poster clashed with the shop's branding and did not display it [ID01]. Another owner was highly critical stating that *"posters that big look stupid"* and suggested a preference for alternative promotional material (e.g. leaflet), detailing the *"benefits of buying smaller"* [ID06].

For peer review only - http://bmjopen.bmj.com/site/about/guidelines.xhtml

Importance of quality customer service

All respondents stressed the importance of high quality customer service to delivering smaller portion meals. One owner who had been involved in the Fish & Chip industry *"Pretty much all my life"* [ID09], had implemented numerous changes following the intervention with support from an owner who spoke at the engagement event. One owner who had been unable to attend the event, reported that they had not implemented smaller portion meals in the evening as they could not rely on serving staff to deliver these consistently [ID05].

Customer feedback

Few respondents reported receiving feedback on smaller portion meals from customers. However, one detailed the enthusiasm from a group of builders who had seen the posters and welcomed the change [ID09].

The ease of recording sales data

Those who provided sales data, reported that this had been straight forward. However, till systems limited the value of these for analyses.

Interviews with those who developed and delivered the intervention

Interviews were conducted with three people from HC. Thematic analysis identified five themes. In order to retain anonymity, the quotes below are not specifically attributed to a given intervention deliverer.

For peer review only - http://bmjopen.bmj.com/site/about/guidelines.xhtml

Intervention deliverers' motivations regarding intervention delivery

HC representatives viewed individual shops as focused on daily sales meaning it was up to HC to take *"strategic long-term views of the industry"*. Respondents wanted shops to succeed in an increasingly competitive marketplace. They took responsibility for driving sector innovation and acknowledged that *"I need Fish & Chip Shops to stay in business because they pay my wages"*.

Considerations in development of the intervention

HC representatives stated that effective engagement on smaller portion sizes with owners/managers could only be achieved by highlighting the financial and customer service benefits. HC staff saw themselves as providing information and choice to their customers, *"It is up to the customer [business owner] to make that choice, we are not going to force them to do anything"*. They were also happy to take the lead on intervention development and delivery and the cost to HC was viewed as an *"investment"*.

Intervention deliverers' views on acceptability

Representatives of HC were disappointed with attendance at the engagement event (9 of 31) and were frustrated that some shops *"didn't realise the potential"*. While many had not provided a reason for non-attendance, some reportedly told HC that it was due to staffing issues. However, the responses HC received from those who did attend were positive, *"I had quite a lot of people ring up and thanking me for the event"*, and they had viewed it as a rare *"interactive"* event: *"Fish fryers in the same room sharing ideas and you could see people writing down notes and bringing up their own problems. Other people were listening, engaging and offering advice and help. That doesn't happen often enough"*.

For peer review only

HC staff were aware that not all businesses would display the posters, with some owners/managers reporting that they were too big. However, they felt that it was important to provide the means to clearly distinguish between regular and smaller portion meals.

HC also viewed the incentive component of the intervention as an act of *"goodwill"* showing their commitment to the intervention. They did not see incentives as imperative to owner/manager involvement and, indeed, not all shops took advantage of them.

Future plans

As a direct result of this work, HC staff developed specific packaging for smaller portion meals and associated promotional material. At the time of interviews they were also trying to source smaller fish fillets for this new packaging.

While HC staff saw smaller portion packaging as a sustainable change, more sustainable methods of delivering the portion control message were required. The engagement event and one-to-one visits were not considered scalable or efficient, *"I can't go around and visit thousands of Fish & Chip Shops because I am only one person"*.

Experience of working with the research team

HC staff found working with the research team a positive and *"enjoyable experience"*. It had *"re-stimulated our [HC's] efforts"* and was felt to be rewarding for both parties.

For peer review only - http://bmjopen.bmj.com/site/about/guidelines.xhtml

DISCUSSION

Statement of principal findings

We found it was feasible to co-design and deliver an intervention to promote smaller portions with a commercial partner and the intervention was acceptable to both Fish & Chip Shops and their customers. Attendees at the engagement event valued and enjoyed it. Shop owners/managers were broadly willing to introduce and promote smaller portion meals. We successfully measured portions sizes and collected some sales data. We observed a reduction in the size of both regular and smaller portion meals after the intervention and an increase in the proportion of meals sold that were a smaller portion. The reduction in portion size of regular meals was due to reduction in chips – the least nutrient-dense component of the meal. As all participating owners/managers considered that their businesses provided smaller portion meals in some form at baseline, the additional overt promotion was broadly acceptable. Most also used box packaging at baseline meaning introduction of this was unlikely to be a key component of the intervention. The evaluation was conducted independently, avoiding the potential for competing interests of the commercial partners.

Strengths and limitations of the study

To our knowledge, this is the first study to evaluate the feasibility of working with a wholesale supplier to co-design and deliver a public health intervention, and to demonstrate the potential role of wholesale suppliers in improving the food offerings from hot food takeaways.

Covert observations, while feasible, would be resource intensive in a larger study. While participating shops had high FSA hygiene ratings,[27] they covered a wide range of IMD deciles,[26] suggesting the intervention may be feasible across a range of socio-economic settings. Acceptability to shops with lower hygiene ratings is unknown. Participants did not feel goal-setting through the

For peer review only - http://bmjopen.bmj.com/site/about/guidelines.xhtml

‘public pledge’ was useful. Greater clarity concerning what was expected of shop owners/managers may have improved this. Some shops did not make use of posters and other marketing materials, such as leaflets, may have engaged a wider range of shops. We did not conduct in-depth interviews with customers and these would provide more insight into their choices and preferences. Some aspects of the intervention were not felt to be sustainable by HC and further thought is required to determine how any such intervention could be scaled-up. We collected no data on customers’ total diets or total population impact.

Due to time and resource constraints our data is unlikely to be representative. Furthermore, we did not reach data saturation in interviews, nor was our customer survey validated or piloted prior to use. Our findings may not be generalisable beyond the UK.

Strengths and limitations in relation to other studies

There are a limited number of intervention studies targeting takeaways in England, and few have been evaluated.[23] Most interventions to date have been delivered by local authorities, limiting their geographical reach. Suppliers, such as HC, have a much greater geographical reach. While mandatory approaches to portion control may be more effective than voluntary schemes,[30, 31] these may be harder to implement.[32, 33] Our intervention is a rare voluntary, industry-led approach to portion control. Difficulties engaging independent takeaways in public health interventions have been previously described, where simply identifying a given takeaway owner can be challenging.[17] The 29% recruitment rate we achieved compares favourably to other interventions in the sector; in another, unpublished, local authority-led study we achieved a 10% recruitment rate.[34] We received limited feedback from those who did not attend the engagement event, although lack of staff cover may be a problem.

For peer review only - http://bmjopen.bmj.com/site/about/guidelines.xhtml

Study implications

Our findings suggest that, within the takeaway sector, it is feasible to develop a supplier-led intervention based on ‘providing information’ and ‘enabling choice’[35] and that this is acceptable across stakeholders. We highlight the importance of product specific packaging that constrains portion size, which can, in part, offset variability in servers’ ability to deliver consistent portions. HC’s smaller portion box packaging was designed and branded to deliver a smaller sized ‘Lite-BITE®’ meal.[36] Sales of these boxes provide evidence of longer-term viability; in 2017 HC sold 552,300 units of the ‘Lite-BITE®’ boxes to 253 unique accounts [D. McLean, personal communication, 2018]. Takeaway owners/managers seem likely to be more receptive to messages about portion control from peers than external organisations, framed primarily in the context of the potential financial benefits. The individual responsible for implementing changes in a takeaway (usually the owner or manager) may require clear and practical instructions on how to make changes. Ideally, interventions should seek to engage with takeaway owners, or those responsible for branding, and this person should communicate changes to serving staff, within the wider context of good ‘customer service’.

Unanswered questions and future research

HC are not planning further engagement events. An alternative platform to deliver the information and guidance in a collective format may be required to maximise the potential of smaller portion packaging (e.g. seminars at trade events). While the promotion of smaller portion meals was broadly acceptable, over half of the customers surveyed, that had not previously purchased the smaller portion meals, were not interested in trying in the future. However, smaller portion meals were clearly attractive to others. Further work is required to assess whether and how customer choices can be further changed.[35] Qualitative interviews with customers could usefully inform this,

For peer review only - http://bmjopen.bmj.com/site/about/guidelines.xhtml

inclusive of their views with regards to meal value-for-money. Future research could explore the impact of smaller portion meals in Fish & Chip Shops on customers' diet and the wider public health implications, as well as the potential to promote smaller meal portions through trade organisations and their events. Owners and managers would additionally benefit from a clearer definition of what constitutes a smaller portion meal from a practical perspective. Defining and developing guidelines to support delivery would be of use.

While this intervention was feasible in a sample of Fish & Chip Shops, further work should seek to identify other sectors of the takeaway and wider catering industry where such an approach could be applied.

ETHICS

The study received ethical approval by the Newcastle University Research Ethics Committee. All Fish & Chip Shop owners/managers and those from HC who delivered the intervention provided written informed consent prior to participation. All customers who participated in the customer survey were provided with study information and provided oral consent prior to participation. Owners/managers were not required to give specific consent for the covert observation component of the data collection.

ACKNOWLEDGEMENTS

We thank Duncan McLean (director) and Bill Colbeck (chairman) of Henry Colbeck Limited for their time and commitment of resources to delivering the intervention, as well as their patience, understanding and respect of the study's research process. We would also like thank Laura Cutler,

For peer review only - http://bmjopen.bmj.com/site/about/guidelines.xhtml

Eimear Duffy, Joel Halligan, Eimear Mullen and Maisie Rowland from the Human Nutrition Research Centre, Newcastle University who provided invaluable help with data collection.

FUNDING

This paper presents independent research funded by the National Institute for Health Research (NIHR) School for Public Health Research (SPHR). The views expressed in this publication are those of the author(s) and not necessarily those of the NHS, NIHR or the Department of Health. The SPHR was not involved in the design of the study, analysis, interpretation of data nor writing the manuscript.

This research was part of the SPHR funded project: *Transforming the 'foodscape': development and feasibility testing of interventions to promote healthier takeaway, pub or restaurant food,* with additional support from Durham and Newcastle Universities, SPHR is funded by the National Institute for Health Research (NIHR). At the time of funding SPHR was a partnership between the Universities of Sheffield, Bristol, Cambridge, Exeter, University College London; The London School for Hygiene and Tropical Medicine; the LiLaC collaboration between the Universities of Liverpool and Lancaster; and Fuse, the Centre for Translational Research in Public Health, a collaboration between Newcastle, Durham, Northumbria, Sunderland and Teesside Universities. Authors LG, FHB, NH, VAS, LP, CDS, AAL and AA are members of Fuse. Funding for Fuse comes from the British Heart Foundation, Cancer Research UK, Economic and Social Research Council, Medical Research Council, the National Institute for Health Research, under the auspices of the UK Clinical Research Collaboration, and is gratefully acknowledged. AA is funded by the NIHR as a NIHR Research Professor. JA and MWh are funded by the Centre for Diet and Activity Research (CEDAR), a UKCRC Public Health Research Centre of Excellence. Funding from the British Heart Foundation, Cancer Research UK, Economic and Social Research Council, Medical Research Council, the National Institute

For peer review only

For peer review only - http://bmjopen.bmj.com/site/about/guidelines.xhtml

for Health Research, and the Wellcome Trust, under the auspices of the UK Clinical Research Collaboration, is gratefully acknowledged.

COMPETING INTERESTS

The research funding contributed to the cost of design and production of some of the intervention materials. HC also contributed to the costs of intervention development and funded delivery. HC developed and sell the 'Lite-BITE®' Fish & Chip meal boxes detailed in the study. Neither the project, nor individual researchers, received financial contributions from HC for this study or any other work.

MWh is funded by NIHR as Director of its Public Health Research Funding Programme. AA is funded by NIHR as a Research Professor and the National Director of NIHR SPHR. CS and MWh are principal investigators in the NIHR SPHR. MWo led and delivered the intervention and provided the packaging materials; at the time of the research he was an undergraduate student at Newcastle Business School, Northumbria University on secondment to HC. After completion of the study (and his degree programme at Northumbria University), he became an employee of HC.

Henry Colbeck Limited

HC an independent supplier to over 2,500 Fish & Chip Shops in the North-East of England, Yorkshire, Cumbria and Scotland. HC is a private limited company founded in 1893 and based in Gateshead, North-East England. Company number 00822749 (www.colbeck.co.uk).

For peer review only

DATA SHARING STATEMENT

Anonymised data relating to the covert observations, sales of Fish & Chip meals, customer survey and semi-structured interviews are available on reasonable request from the corresponding author (LG).

AUTHOR CONTRIBUTIONS

JA, VAS, CDS, AAL, MWh & AA devised the concept for the Foodscape project. MWo developed the intervention with support from LG, FHB, VAS and AA. The evaluation study and associated methods were designed by LG, FHB, JA, VAS, LP, WW, CDS, MWh and AA. Data collection was overseen by LG and completed by LG, MWo, FHB, NH and AA. LG led on data analysis and drafting the manuscript, supported by MWh and AA. All authors provided critical comments on drafts of the manuscript, and read and approved the final version.

PATIENT INVOLVEMENT

No patients were involved in the study.

COPYRIGHT STATEMENT

The promotional posters are copyrighted to Henry Colbeck Limited and cannot be reproduced without their expressed permission.

For peer review only

LEGENDS:

Table 1 - Summary of data collected

Table 2 - Summary of covert observation data collected from each shop

Table 3 - Customer survey responses

Figure 1 - Promotional A0 size poster options

Figure 2 - Portion control intervention participation and data collection flowchart

Supplementary File A - Logic model

Supplementary File B - Goal-setting form

Supplementary File C - TIDieR checklist

Supplementary File D - Customer survey

REFERENCES

1. Jaworowska A, M. Blackham T, Long R, et al. Nutritional composition of takeaway food in the UK. *Nutrition & Food Science* 2014;44(5):414-30. doi: doi:10.1108/NFS-08-2013-0093

2. Lachat C, Nago E, Verstraeten R, et al. Eating out of home and its association with dietary intake: a systematic review of the evidence. *Obesity Reviews* 2012;13(4):329-46. doi: 10.1111/j.1467-789X.2011.00953.x

3. Public Health England. Calorie reduction: The scope and ambition for action. In: Department of Health and Social Care, ed., 2018.

4. Goffe L, Rushton S, White M, et al. Relationship between mean daily energy intake and frequency of consumption of out-of-home meals in the UK National Diet and Nutrition Survey. *International Journal of Behavioral Nutrition and Physical Activity* 2017;14(1):131. doi: 10.1186/s12966-017-0589-5

For peer review only

For peer review only - http://bmjopen.bmj.com/site/about/guidelines.xhtml

5. Pereira MA, Kartashov AI, Ebbeling CB, et al. Fast-food habits, weight gain, and insulin resistance (the CARDIA study): 15-year prospective analysis. *The Lancet* 2005;365(9453):36-42. doi: http://dx.doi.org/10.1016/S0140-6736(04)17663-0

6. Bodicoat DH, Carter P, Comber A, et al. Is the number of fast-food outlets in the neighbourhood related to screen-detected type 2 diabetes mellitus and associated risk factors? *Public Health Nutrition* 2014;FirstView:1-8. doi: doi:10.1017/S1368980014002316

7. Walton JK. Fish and Chips, and the British Working Class, 1870-1940: A&C Black 1994.

8. Brooks J. Fish and Chips in Commercial Foodservice 2016: Seafish, 2017.

9. Seafish, AHDB Potatoes, Campden BRI. Enjoy Fish and Chips - Synopsis report. enjoyfishandchips.co.uk, 2016.

10. Scientific Advisory Committee on Nutrition. Dietary Reference Values for Energy: The Stattionery Office London, 2011.

11. Hollands GJ, Shemilt I, Marteau TM, et al. Portion, package or tableware size for changing selection and consumption of food, alcohol and tobacco. *Cochrane Database of Systematic Reviews* 2015(9) doi: 10.1002/14651858.CD011045.pub2

12. Marteau TM, Hollands GJ, Shemilt I, et al. Downsizing: policy options to reduce portion sizes to help tackle obesity. *BMJ* 2015;351 doi: 10.1136/bmj.h5863

13. Steenhuis I, Poelman M. Portion Size: Latest Developments and Interventions. *Current Obesity Reports* 2017;6(1):10-17. doi: 10.1007/s13679-017-0239-x

14. Hollands GJ, Cartwright E, Pilling M, et al. Impact of reducing portion sizes in worksite cafeterias: a stepped wedge randomised controlled pilot trial. *International Journal of Behavioral Nutrition and Physical Activity* 2018;15(1):78. doi: 10.1186/s12966-018-0705-1

15. Sharpe R. An inconvenient sandwich: the throwaway economics of takeaway food: New Economics Foundation 2010.

For peer review only

16. Bagwell S, O'Keefe E, Doff S, et al. Encouraging healthier takeaways in low-income communities: Tools to support those working to encourage healthier catering amongst fast food takeaways. London: Cities Institute, London Metropolitan University, 2014.

17. Goffe L, Penn L, Adams J, et al. The challenges of interventions to promote healthier food in independent takeaways in England: qualitative study of intervention deliverers' views. *BMC Public Health* 2018;18(1):184. doi: 10.1186/s12889-018-5096-3

18. Henry Colbeck Limited. Henry Colbeck Limited: About Us Gateshead2016 [Available from: http://www.colbeck.co.uk/about-us accessed Accessed October 2017.

19. Adams J, Goffe L, Brown T, et al. Frequency and socio-demographic correlates of eating meals out and take-away meals at home: cross-sectional analysis of the UK national diet and nutrition survey, waves 1-4 (2008-12). *International Journal of Behavioral Nutrition and Physical Activity* 2015;12(1):51.

20. Goffe L, Hillier-Brown F, Doherty A, et al. Comparison of sodium content of meals served by independent takeaways using standard versus reduced holed salt shakers: cross-sectional study. *International Journal of Behavioral Nutrition and Physical Activity* 2016;13(1):102. doi: 10.1186/s12966-016-0429-z

21. Goffe L, Wrieden W, Penn L, et al. Reducing the Salt Added to Takeaway Food: Within-Subjects Comparison of Salt Delivered by Five and 17 Holed Salt Shakers in Controlled Conditions. *PLOS ONE* 2016;11(9):e0163093. doi: 10.1371/journal.pone.0163093

22. Hillier-Brown FC, Summerbell CD, Moore HJ, et al. The impact of interventions to promote healthier ready-to-eat meals (to eat in, to take away or to be delivered) sold by specific food outlets open to the general public: a systematic review. *Obesity Reviews* 2016:n/a-n/a. doi: 10.1111/obr.12479

23. Hillier-Brown FC, Summerbell CD, Moore HJ, et al. A description of interventions promoting healthier ready-to-eat meals (to eat in, to take away, or to be delivered) sold by specific food

For peer review only

outlets in England: a systematic mapping and evidence synthesis. *BMC Public Health* 2017;17(1):93. doi: 10.1186/s12889-016-3980-2

24. Michie S, Richardson M, Johnston M, et al. The behavior change technique taxonomy (v1) of 93 hierarchically clustered techniques: building an international consensus for the reporting of behavior change interventions. *Annals of behavioral medicine : a publication of the Society of Behavioral Medicine* 2013;46(1):81-95. doi: 10.1007/s12160-013-9486-6 [published Online First: 2013/03/21]

25. Hoffmann TC, Glasziou PP, Boutron I, et al. Better reporting of interventions: template for intervention description and replication (TIDieR) checklist and guide. *BMJ : British Medical Journal* 2014;348 doi: 10.1136/bmj.g1687

26. Department for Communities and Local Government. English indices of deprivation 2015. In: UK Government, ed., 2015.

27. Food Standards Agency. Food hygiene ratings 2018 [updated Accessed March 2018. Available from: http://ratings.food.gov.uk/ accessed 20/7/2015 2015.

28. R: A Language and Environment for Statistical Computing [program]: R Foundation for Statistical Computing, 2018.

29. Ritchie J, Lewis J, Nicholls CM, et al. Qualitative research practice: A guide for social science students and researchers: Sage 2013.

30. Lloyd-Williams F, Bromley H, Orton L, et al. Smorgasbord or symphony? Assessing public health nutrition policies across 30 European countries using a novel framework. *BMC Public Health* 2014;14(1):1195. doi: 10.1186/1471-2458-14-1195

31. McGill R, Anwar E, Orton L, et al. Are interventions to promote healthy eating equally effective for all? Systematic review of socioeconomic inequalities in impact. *BMC Public Health* 2015;15(1):457. doi: 10.1186/s12889-015-1781-7

32. Department of Health. Localising the Public Health Responsibility Deal: Toolkit for Local Authorities, 2013.

For peer review only

For peer review only - http://bmjopen.bmj.com/site/about/guidelines.xhtml

33. Knai C, Petticrew M, Durand MA, et al. Has a public–private partnership resulted in action on healthier diets in England? An analysis of the Public Health Responsibility Deal food pledges. *Food Policy* 2015;54:1-10. doi: http://dx.doi.org/10.1016/j.foodpol.2015.04.002

34. Hillier-Brown F, Lloyd S, Muhammad L, et al. Feasibility and acceptability of a Takeaway Masterclass aimed at improving healthier cooking practices and menu options in takeaway food outlets. *In Submission* 2018

35. Nuffield Council on Bioethics. Public health: ethical issues: Nuffield Council on Bioethics 2007.

36. Henry Colbeck Limited. The Lite-BITE® Gateshead2016 [Available from: http://www.colbeck.co.uk/sf-docs/resources/Lite-bitewebpage.pdf accessed Accessed March 2017.

For peer review only

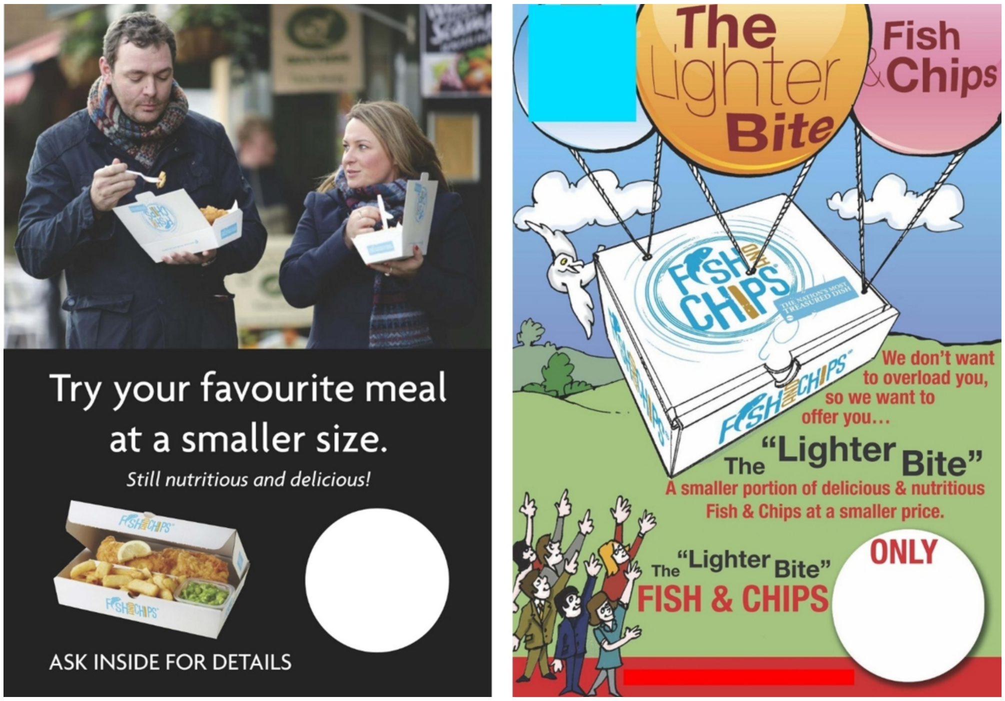

Figure 1. Promotional A0 size poster options

275x190mm (300 x 300 DPI)

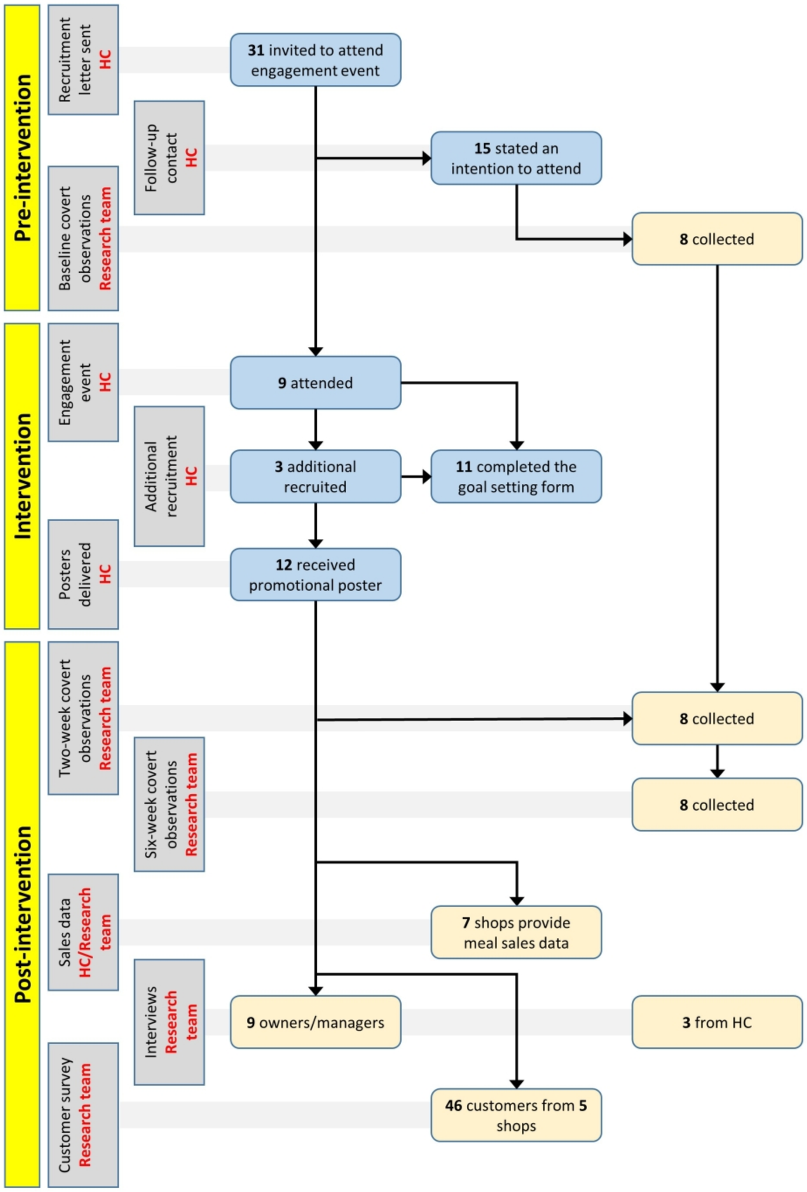

Figure 2. Portion control intervention participation and data collection flowchart

190x275mm (300 x 300 DPI)

Supplementary File A: Logic model

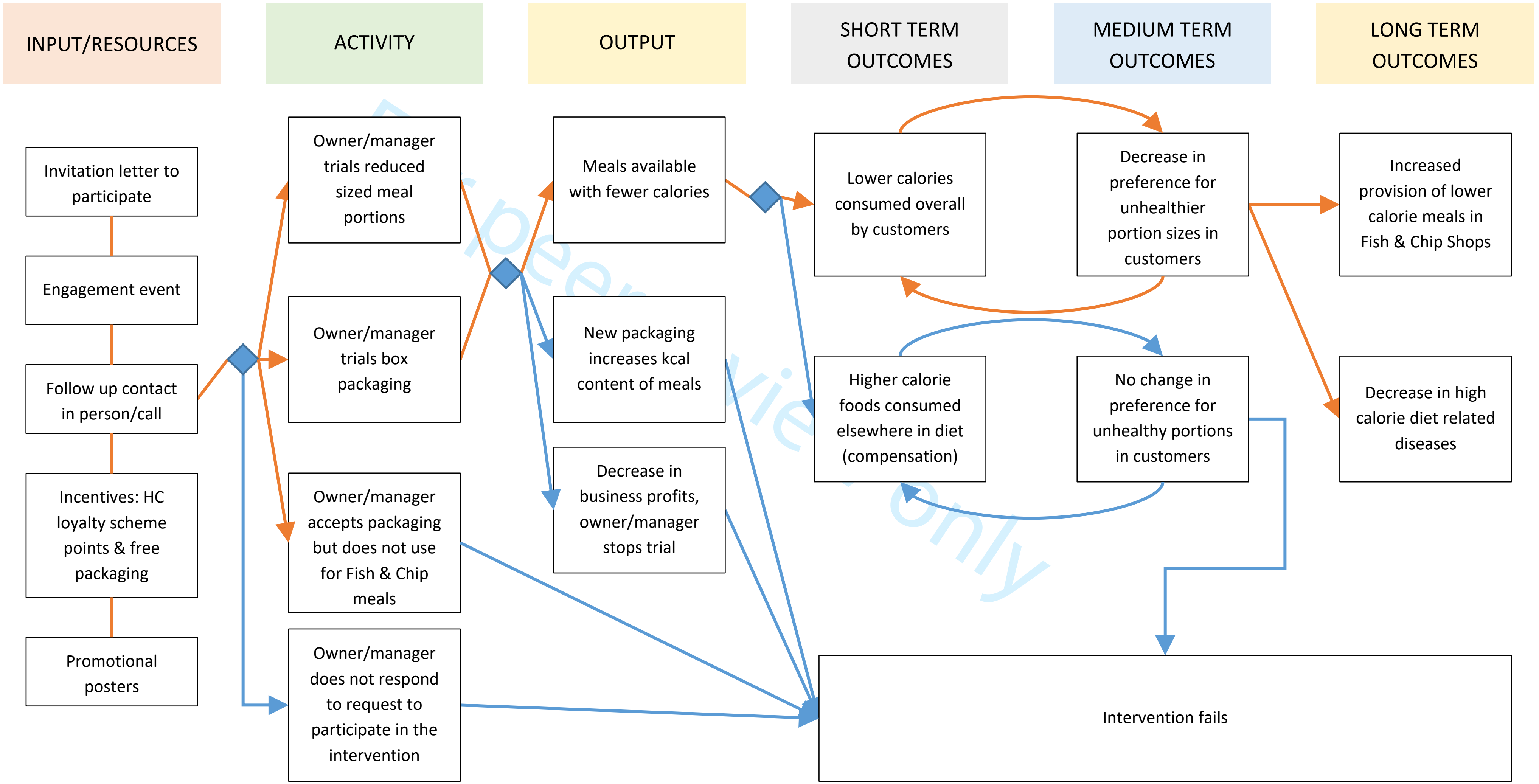

Supplementary File B: Goal-setting form

Team Spirit Event, Henry Colbeck Limited,

Wednesday 6th April 2016

Business name: ____

Business representatives:

Name: ______________________________________ Role: owner/ manager/ staff (circle answer)

Name: ______________________________________ Role: owner/ manager/ staff (circle answer)

From the topics covered at this Team Spirit event; what are the things you could change in your business?

	1. Could your business make this change? (Y/N)	2. Are you willing to try this change? (Y/N)
**Change to structured packaging:** *e.g. introduce bio boxes*		
**Make smaller portions available to your customers at *all times***		

Public Pledge: *Detail what, which and when you will change*

**What to change?** *Structured packaging /Smaller portions*	**Which packaging will you use for which change?** *Single compartment corrugated cardboard box/Double compartment corrugated cardboard box/Bio box/MK*	**When will you do it?** *Date*	**How sure (confident) are you that your business can achieve this?** *From 1-5 (1=not at all sure; 5= very sure)**
1.			
2.			

*1= not at all sure; 2= not very sure; 3= neither; 4= somewhat sure; 5= very sure

I give consent to be contacted both, by phone, or in person by Newcastle University to provide them with details of the:

- Packaging changes I have made (if any)
- The number of Fish & Chips portions (all sizes) sold before and after the project
- My comments and opinions of the Team Spirit event
- My comment and opinions of any changes I have made following the event

Additionally, I will allow Newcastle University to speak with a few of my customers to hear their thoughts.

Signed________________________ Print Name___________________________

Phone Number (and best time to call) ______________________________

For peer review only

Packaging key:

- Corrugated cardboard boxes are available in either single or double compartment versions. The double compartment boxes provides separate compartments for each of the Fish & Chip meal components.
- The Bio box packaging are a single compartment box constructed from biodegradable material extracted from sugar cane.
- MK packaging are a single compartment box constructed of non-corrugated food grade cardboard.

For peer review only

Supplementary File C: TIDieR checklist

Description of the portion control engagement event using the template for intervention description and replication (TIDieR) checklist.[1]

1. Name [page 8]	Engagement event – Henry Colbeck Ltd
2. Rationale, theory, goal [page 8]	Fish & Chip meals from takeaways are generally characterised by large portion sizes.[2] People who eat more meals away from home tend to eat more calories per day [3] and consumption of takeaway meals has been linked to weight gain [4] and diet-related diseases.[5] A systematic review revealed that a person consumes more when presented with a larger-sized portion.[6]<br><br>The behaviour change techniques [7 8] used during the training were:<br>• Providing information on behavioural outcomes<br>• Instruction on how to perform the behaviour<br>• Demonstration of behaviour/credible source/vicarious consequences<br>• Information about others' approval<br>• Pros and cons<br>• Behavioural practice/rehearsal<br>• SMART goal setting (behaviour) and action planning<br>• Behavioural contract<br>• Adding objects to the environment<br>• Self-monitoring of behaviour (sales)<br>• Monitoring of behaviour by others without feedback<br>• Reward (outcome)/Material Incentive<br>• Financial incentive/form for specific goods [8]<br><br>Aim of the intervention: To increase the provision and promotion of smaller portion meals available to all customers throughout all opening hours and/or increased/renewed focus on portion control across all meals.
3. Materials [page 8]	Information provision on the consequences of behaviour and behaviour change opportunities, presented by owners of established Fish & Chip Shops and the supplier.<br><br>Owners, managers and their staff of Fish & Chip Shops that attended were presented with details, primarily financial, of the benefits of effective portion control and the promotion and provision of smaller portion meals. These were predominantly in the form of PowerPoint presentations, but also included further visual aids such as the displaying of a locally purchased Fish & Chips meal on an 11 inch plate to illustrate the volume of food delivered.

For peer review only

	Participants were asked to read and sign a 'public pledge' as part of the goal setting form that stipulated which proposed activities they could and would be willing to make in their business.<br><br>Owners/managers were offered, by the supplier, two incentives to support fulfilment of the pledges. These were loyalty scheme points (financial) and a free case (100 units) of box packaging of their choosing (specific goods). Additionally, they were offered free posters to promote the availability of the smaller portion meals to the shop's customers.<br><br>The research team also provide a booklet to record Fish & Chip meal sales during the evaluation period to monitor sales behaviour.<br><br>A free lunch was included as part of the engagement event for all those that attended.
4. Procedures [page 8]	Fish & Chip Shop owners/managers were invited via a postal communication from the supplier one month prior to the engagement event.<br><br>On the day of the event, participants were invited to a hotel in close proximity (under 3km) to Henry Colbeck Ltd's office and warehouse facility.<br><br>There was a range of presentations delivered from both the perspective of the supplier, Henry Colbeck Ltd and Fish & Chip business owners, as well as study data collection requirements from the research team (LG).<br><br>Henry Colbeck Ltd's presentations set out the reasons for initiating the event, and contextualised existing portion sizes in the Fish & Chip industry in relation to both an adult's diet as well as other sectors of the fast-food industry. They detailed the packaging available to support improved portion control and smaller meal portion delivery. They also stated the items that they would provide to incentivise shop participation.<br><br>One business owner (Owner A) detailed his experience of delivering highly regulated portion sizes, as well as the benefits to his business of providing a range of smaller portion meals. This included knowing one's target portion size, selecting the appropriate packaging, staff training, sales and profit margins of smaller meals. A second business owner (Owner B) detailed the experience of the implementation and financial success within his shop of smaller portion meals.<br><br>LG detailed what data the research team intended to collect as part of the evaluation.<br><br>Following the presentations, participants were provided with lunch and refreshments, followed by a question and answer session before the close.

For peer review only

	Promotional material was provided through the sales team at Henry Colbeck Ltd and owners/managers ordered the free case of packing using their usual method of ordering through Henry Colbeck Ltd.
5. Who provided [page 8]	The educational material delivered by Henry Colbeck Ltd were developed by themselves in conversation with the research team. Additionally, Owner A, created his own presentation material and Owner B, presented without supporting material.<br><br>The goal setting, action planning and pledge form (behavioural contract) was developed by the research team, guided by VAS, health psychology expert. The research team provided all material to enable participants to record sales data in the required format.
6. How [page 8]	Training delivered in person in a group session.
7. Where [page 8]	Hotel, in close proximity to Henry Colbeck Ltd's office and warehouse facility, Gateshead.
8. When and how much [page 8]	One session approximately three hours.
9. Tailoring [page 8]	The material provided were tailored specifically to Fish & Chips Shops that offered a takeaway service.
10. Modifications [page 8]	This was the first time the intervention had been delivered. However, previous material circulated by Henry Colbeck Ltd to its customers had been adapted and incorporated accordingly to the setting of delivering the information 'in-person'.
11. Planning/fidelity (ways to maintain fidelity) [page 8]	Members of the research team attended the engagement event to assess if it was delivered as intended. As the event was a one-off no strategies were implemented to maintain or improve fidelity.
12. Actual fidelity [page 8]	All presentations were delivered as intended. However, the goal setting activity was not. Participants had little time to complete the form and no support. During follow-up interviews many could not recall completing this or had not seen any value in the pledge.

References

1. Hoffmann TC, Glasziou PP, Boutron I, et al. Better reporting of interventions: template for intervention description and replication (TIDieR) checklist and guide. BMJ : British Medical Journal 2014;**348** doi: 10.1136/bmj.g1687[published Online First: Epub Date]|.

2. Jaworowska A, M. Blackham T, Long R, et al. Nutritional composition of takeaway food in the UK. Nutrition & Food Science 2014;**44**(5):414-30 doi: doi:10.1108/NFS-08-2013-0093[published Online First: Epub Date]|.

3. Goffe L, Rushton S, White M, et al. Relationship between mean daily energy intake and frequency of consumption of out-of-home meals in the UK National Diet and Nutrition Survey. International Journal of Behavioral Nutrition and Physical Activity 2017;**14**(1):131 doi: 10.1186/s12966-017-0589-5[published Online First: Epub Date]|.

For peer review only

4. Pereira MA, Kartashov AI, Ebbeling CB, et al. Fast-food habits, weight gain, and insulin resistance (the CARDIA study): 15-year prospective analysis. The Lancet 2005;**365**(9453):36-42 doi: http://dx.doi.org/10.1016/S0140-6736(04)17663-0[published Online First: Epub Date]|.

5. Bodicoat DH, Carter P, Comber A, et al. Is the number of fast-food outlets in the neighbourhood related to screen-detected type 2 diabetes mellitus and associated risk factors? Public Health Nutrition 2014;**FirstView**:1-8 doi: doi:10.1017/S1368980014002316[published Online First: Epub Date]|.

6. Hollands GJ, Shemilt I, Marteau TM, et al. Portion, package or tableware size for changing selection and consumption of food, alcohol and tobacco. Cochrane Database Syst Rev 2015;**9**:CD011045 doi: 10.1002/14651858.CD011045.pub2[published Online First: Epub Date]|.

7. Michie S, Richardson M, Johnston M, et al. The behavior change technique taxonomy (v1) of 93 hierarchically clustered techniques: building an international consensus for the reporting of behavior change interventions. Annals of behavioral medicine : a publication of the Society of Behavioral Medicine 2013;**46**(1):81-95 doi: 10.1007/s12160-013-9486-6[published Online First: Epub Date]|.

8. Adams J, Giles EL, McColl E, et al. Carrots, sticks and health behaviours: a framework for documenting the complexity of financial incentive interventions to change health behaviours. Health psychology review 2014;**8**(3):286-95 doi: 10.1080/17437199.2013.848410[published Online First: Epub Date]|.

For peer review only

Goffe et al. 2018. *Feasibility of working with a wholesale supplier to co-design and test acceptability of an intervention to promote smaller portions: an uncontrolled before-and-after study in British Fish & Chip Shops*

Customer survey

Interviewee ID	
Business name	
Date	

Fish and chip shop use

1. Do you use this fish and chip shop regularly?

Yes	No
☐	☐

2. What do you mean by regularly?

More than once a week	Once a week	Once every 2 weeks	Once a month	Once every 3 months	Once every 6 months	Once a year	First time
☐	☐	☐	☐	☐	☐	☐	☐

3. What are the main two reasons why you come to this fish and chip shop?

Taste/Quality	Convenience	Price	Portion size
☐	☐	☐	☐

Purchase behaviour

4. For how many people do you usually order for?

Write answer:

5. What do you normally order and what portion size? i.e. large/regular/smaller

Write answer:

Portion size

6. What do you think of the portion sizes here?

Too small	Just right	Too big
☐	☐	☐

Smaller portions

7. Are you aware of the smaller portions offered?

Yes	No
☐	☐

For peer review only

Goffe et al. 2018. *Feasibility of working with a wholesale supplier to co-design and test acceptability of an intervention to promote smaller portions: an uncontrolled before-and-after study in British Fish & Chip Shops*

8. Have you noticed the new posters or other forms of promotion in the shop?

Yes	No
☐	☐

9. Have you tried the smaller portions?

Yes	No
☐	☐

10. If not, would you buy it in future?

NA	Yes	No
☐	☐	☐

11. If yes, who for?

Write answer:

Other options

12. Are there any meals/dishes/portion sizes you would like to see offered at this fish and chip shop?

Write answer:

Gender

Female	Male
☐	☐

Age category

18 – 30	31 - 40	41 - 50	51 - 60	61 - 70	Over 70
☐	☐	☐	☐	☐	☐

For peer review only

Goffe et al. 2018. *Feasibility of working with a wholesale supplier to co-design and test acceptability of an intervention to promote smaller portions: an uncontrolled before-and-after study in British Fish & Chip Shops*

Owner/manager interview topic guide

Interviewee background

- Please could you introduce yourself and state your role in the business
- Do you have any other premises?
- How long have you been working in fish and chips?
- How did you get into fish and chips?
- What is special or unique about your business?

Engagement with the project

- Did you attend the engagement event?
    - Why did you decide to attend the event?
    - What did you think about the event?
        - Prompts:
            - Speakers
            - Material
            - Location, i.e. convenience
            - Venue & refreshments
            - Henry Colbeck and their staff
            - Information delivered
    - What did you like?
    - Did you learn anything new?
    - Did you speak with other owners?
    - Any missing stakeholders?
    - Was there any part of the event you thought was missing, disliked or would change?
    - Was it worthwhile attending?
- If you didn't attend, did you speak with Henry Colbeck about portion control?
    - What did they tell you about portion control?
    - Was any of the information new?
    - What changes did they ask you to make?
    - What made you choose to give the posters/smaller portions a go?
    - Anything further at the time of the visit that could have been offered or suggested to support you to make changes?
- Did you complete a pledge sheet/Do you remember the pledge sheet?

For peer review only

Goffe et al. 2018. *Feasibility of working with a wholesale supplier to co-design and test acceptability of an intervention to promote smaller portions: an uncontrolled before-and-after study in British Fish & Chip Shops*

Changes

- What changes have you made since attending the event?
    - Prompts
        - Increased awareness/regulation of portion control in your staff
        - Reduced portion sizes
        - Changed packaging
        - Introduced smaller portions
        - Put up poster(s)
- What's the role/importance of customer service?
- What response from customers?
    - Prompts
        - Changes in sales increase/decrease
        - Comments/Conversations
- Have you gained new customers?

Posters

- Where you offered both poster designs?
- Did you display them? And where? (possibly answered from previous questions)
    - If not, why not
    - If only inside/outside why?
- What did you think about the posters?
    - Prompts
        - Design
        - Wording
        - Appropriateness for your shop
- Any comments from customers? (possibly answered from previous questions)
- Could you suggest any changes?
- Will you keep the posters up?
- Did you do any further promotion?
    - Prompts
        - Staff led
        - Social media

Incentives

- What did you think of the incentives provided by Henry Colbeck?
    - Prompts
        - Loyalty scheme points
        - Free packaging
- Anything further you feel that they could have offered to support you in offering smaller portions/regulating portion size?

For peer review only

Goffe et al. 2018. *Feasibility of working with a wholesale supplier to co-design and test acceptability of an intervention to promote smaller portions: an uncontrolled before-and-after study in British Fish & Chip Shops*

Sales data

- Did you record sales data?
- Was it easy to keep track of sales data?
    - Prompts
        - Lunchtime/Evening service
- Were the sales booklets that we provided of use?
- Have any of the changes you've made been financially beneficial?

Portion control

- Prior to this engagement with Henry Colbeck was portion control something that you considered?
- As a result of the engagement with Henry Colbeck do you give greater consideration to portion control?
- Is it important to you and your business to offer your customers a range of portion sizes?
- Will you continue to monitor the portion size of your meals?

Future plans

- Are you happy with the changes?
- Will you stick with the changes that you've made?
- Do you plan to make any further changes?

AOB

- Is there anything further you'd like to add about this process?

For peer review only

Goffe et al. 2018. *Feasibility of working with a wholesale supplier to co-design and test acceptability of an intervention to promote smaller portions: an uncontrolled before-and-after study in British Fish & Chip Shops*

Intervention deliverer interview topic guide

Interviewee background

- Please could you introduce yourself and state your role in Henry Colbeck
- How long have you been working for Henry Colbeck?
- What do you feel separates Henry Colbeck from other suppliers in the industry?

Motivation for the work

- History of portion control
  - What work that you know of has been done broadly, what work has Henry Colbeck done specifically?
- Why were you interested in furthering such work (motives)?
  - What does your business stand to gain from such work?
  - Is it purely financial, or other broader enhancement of the industry?
- How did this specific work come to fruition?

Development

- What was your role in the development process?
- What was your role in the delivery process?
- How did you identify suitable project partners and speakers for the event?
- What role did the University research team play in the development of the intervention?
- Incentives, why were they chosen?
- Did it detract from your usual business operations?
- Was it costly to the business?

Engagement event

- Was it delivered as you had envisioned?
- What did you like most about the event?
- Any problems on the day?
- Any feedback from those that attended?
- Anything that you would do differently?

Posters

- Please could you detail the design process regarding the posters?
  - Prompts:
    - Costs
    - Time
    - Stakeholders
    - Designers
- You offered two posters, was there a preference for one over the other?
- Did you receive any feedback from owners?

For peer review only

- Anything learnt for future poster production?

Incentives

- You offered both loyalty scheme points and a free case of packaging, how were they received?
- How important did you feel they were to getting businesses to trial the smaller portions?
- Is there anything else that you considered offering?

Packaging

- You have a wide range of different packaging, was there a particular type you were interested in promoting as part of this work?
- Does the active promotion of specific packaging types raise any issues with customers, for examples those customers that have their own branded packaging?
- Did you receive any useful feedback from your customers?

Your assessment

- Overall how have your customers responded to this work?
- Do you feel that this project has been significant increase in your efforts to promote portion control and smaller portions compared to previous efforts?
- What is your overall assessment of the project? Are you happy? Was this extra and unnecessary work?
- What were the major learning issues for you?
- Did anything surprise you?
- Did you personally enjoy the work?
- Anything you didn’t enjoy or found frustrating?
- Anything that you would do differently?
- What does Henry Colbeck, gain from this work?
- What have you gained personally from this work?

Future plans for smaller portions

- You have continued to the develop packaging for smaller portions, please can you detail what further work you done since the work with the feasibility study
- What was your motivation to continue on with this work?
- How receptive have your customers been?
- Do you have any other future work or ideas regarding portion control or reduced sized portions that you would like to detail?

AOB

- Is there anything further you’d like to add about this process?

For peer review only
